# Bacterial and Viral Coinfections in Adult Patients Hospitalized With COVID-19 Throughout the Pandemic: A Multinational Cohort Study in the EuCARE Project

**DOI:** 10.1093/infdis/jiaf167

**Published:** 2025-04-03

**Authors:** Pontus Hedberg, Karol Serwin, Maria Francesca Greco, Joana P. V. Pereira, Dovile Juozapaite, Sara De Benedittis, Francesca Bai, Nadine Lübke, Tobias Wienemann, Iuri Fanti, Florian König, Nico Pfeifer, Rolf Kaiser, Maurizio Zazzi, Alessandro Cozzi-Lepri, Daniel Naumovas, Giulia Marchetti, Milosz Parczewski, Björn-Erik Ole Jensen, Francesca Incardona, Anders Sönnerborg, Pontus Nauclér

**Affiliations:** Department of Medicine Huddinge, Karolinska Institutet, Stockholm, Sweden; Department of Tropical Infectious Diseases and Immune Deficiency, Pomeranian Medical University in Szczecin, Poland; Department of Health Sciences, Clinic of Infectious Diseases, ASST Santi Paolo E Carlo, University of Milan, Italy; Department of Gastroenterology, Hepatology and Infectious Diseases, Medical Faculty and University Hospital Duesseldorf, Heinrich Heine University, Germany; Vilnius Santaros Klinikos Biobank, Vilnius University Hospital Santaros Klinikos, Lithuania; Department of Health Sciences, Clinic of Infectious Diseases, ASST Santi Paolo E Carlo, University of Milan, Italy; Department of Health Sciences, Clinic of Infectious Diseases, ASST Santi Paolo E Carlo, University of Milan, Italy; Institute of Virology, Medical Faculty and University Hospital Düsseldorf, Heinrich Heine University, Düsseldorf, Germany; Institute of Medical Microbiology and Hospital Hygiene, Medical Faculty and University Hospital Duesseldorf, Heinrich Heine University, Germany; EuResist Network GEIE, Rome, Italy; Institute for Bioinformatics and Medical Informatics and Medical Informatics; Methods in Medical Informatics Department of Computer Science, University of Tübingen; Institute for Bioinformatics and Medical Informatics and Medical Informatics; Methods in Medical Informatics Department of Computer Science, University of Tübingen; Institute of Virology, University of Cologne, Germany; Department of Medical Biotechnologies, University of Siena, Italy; Centre for Clinical Research, Epidemiology, Modelling and Evaluation, Institute for Global Health UCL, London, United Kingdom; Vilnius Santaros Klinikos Biobank, Vilnius University Hospital Santaros Klinikos, Lithuania; VU LSC-EMBL Partnership for Genome Editing Technologies, Life Sciences Center, Vilnius University, Lithuania; Department of Health Sciences, Clinic of Infectious Diseases, ASST Santi Paolo E Carlo, University of Milan, Italy; Department of Tropical Infectious Diseases and Immune Deficiency, Pomeranian Medical University in Szczecin, Poland; Department of Gastroenterology, Hepatology and Infectious Diseases, Medical Faculty and University Hospital Duesseldorf, Heinrich Heine University, Germany; EuResist Network GEIE, Rome, Italy; InformaPRO s.r.l., Rome, Italy; Department of Medicine Huddinge, Karolinska Institutet, Stockholm, Sweden; Department of Clinical Microbiology, Karolinska University Hospital; Department of Clinical Microbiology, Karolinska University Hospital; Division of Infectious Diseases, Department of Medicine, Solna, Karolinska Institutet, Stockholm, Sweden

**Keywords:** antimicrobial stewardship, coinfection, mortality, COVID-19, SARS-CoV-2

## Abstract

**Background:**

Limited evidence exists on how bacterial and viral coinfections have developed since the SARS-CoV-2 Omicron variant emerged. We investigated whether community-onset coinfections in adult patients hospitalized with COVID-19 differed during the wild type, Alpha, Delta, and Omicron periods and whether such coinfections were associated with an increased risk of mortality.

**Methods:**

We conducted a multinational cohort study including COVID-19 hospitalizations until 30 April 2023 in 5 European countries. The outcome was bacterial and viral coinfections based on 5 test modalities. Variant periods were compared with regard to occurrences of coinfections and risk ratios for coinfections (Omicron vs pre-Omicron), as well as association with in-hospital mortality (Omicron vs pre-Omicron).

**Results:**

A total of 29 564 cases were included: 12 601 wild type, 5256 Alpha, 2433 Delta, and 9274 Omicron. The coinfection rate was 2.6% (327/12 601) for wild type, 2.0% (105/5256) for Alpha, 3.2% (77/2433) for Delta, and 7.9% (737/9274) for Omicron. Omicron had a significantly increased risk ratio of coinfection when compared with preceding variants (1.88 [95% CI, 1.53–2.32], *P* < .001). These results were consistent across several subgroup analyses. An increased occurrence (19% [232/1246] vs 11% [3042/28 318]) and adjusted risk (1.69 [95% CI, 1.49–1.91], *P* < .001) of in-hospital mortality were observed in patients with a verified coinfection as compared with patients without a coinfection.

**Conclusions:**

Bacterial and viral coinfections were more prevalent during the Omicron period as compared with preceding variants. Such coinfections were associated with an increased risk of in-hospital mortality, calling for sustained monitoring and clinical vigilance.

The prevalence of bacterial community-onset coinfections among patients hospitalized with COVID-19 is low at approximately 5% [1, 2]. Despite this, high antibiotic use has been observed, causing concerns over antimicrobial stewardship and resistance [3, 4]. However, studies are almost exclusively based on periods preceding the emergence of Omicron, and some studies indicate that coinfections have become more prevalent after Omicron emerged [5–7]. Yet, these studies have been restricted to limited settings and periods or have not contained detailed microbiological data. Therefore, it remains to be understood if the low prevalence of coinfections has remained since Omicron emerged. It is important to understand whether coinfections modify the severity of COVID-19 hospitalizations and how guidelines for empirical treatment with antibiotics should be planned. The pandemic has been associated with changes in circulation of non–SARS-CoV-2 respiratory viruses [8]. This includes profound reductions in circulation of such viruses during the 2020–2021 season, followed by subsequent resurges [9, 10]. A low prevalence of coinfection with influenza in patients with COVID-19 has been reported, but variant-specific estimates and data on other viruses are scarce [11].

In this EuCARE study [12, 13], we aimed to investigate whether the occurrence of bacterial and viral coinfections differed during the Omicron period as compared with preceding variants. Furthermore, we aimed to investigate factors associated with coinfections and if coinfections were associated with an increased risk of mortality.

## METHODS

### Study Design and Data Sources

We conducted a cohort study of bacterial and viral community-onset coinfections in patients hospitalized with COVID-19 in 11 hospitals and 5 sites in Germany, Italy, Lithuania, Poland, and Sweden (Supplementary Figure 1). Data collection was performed retrospectively and prospectively, including electronic health record systems, health registries, and other information systems as previously described [13].

### Study Population

Adults (≥18 years) hospitalized with COVID-19 up to 30 April 2023 were included. COVID-19 hospitalization was defined as testing positive for SARS-CoV-2 by polymerase chain reaction from 14 days before to 2 days after hospital admission. Patients were excluded if they had >2 sequenced SARS-CoV-2 variants or a Beta, Eta, Gamma, or Zeta variant. Patients were also excluded if they were not classified as having a wild type, Alpha, Delta, or Omicron infection due to a lack of a sequenced sample or a national distribution ≤75% for any of these variants [13]. The first hospitalization per patient meeting these criteria was included in the analyses.

### Exposure

The exposure was SARS-CoV-2 variant (wild type, Alpha, Delta, Omicron) based on results from sequencing of the whole genome, multiple genomic regions, or the spike gene; if no sequencing had been performed, it was inferred from the date of hospital admission and country, as previously described [13]. The cutoff used to infer variants was >75% (ie, >75% of GISAID sequences in the specific time window and country belonged to the assigned variant).

### Coinfections and Other Collected Variables

Samples taken from 1 day before to 2 days after hospital admission were considered. Bacterial coinfections included pathogens identified from blood cultures, lower respiratory tract (LRT) cultures, respiratory bacterial DNA assays (*Chlamydophila pneumoniae*, *Chlamydophila psittaci*, *Legionella pneumophila*, *Legionella* species, and *Mycoplasma pneumoniae*), and urinary antigen test (UAT; *Legionella pneumophila* and *Streptococcus pneumoniae*). Viral coinfections included polymerase chain reaction testing of adenovirus, bocavirus, enterovirus, influenza viruses A and B, metapneumovirus, parainfluenza virus, respiratory syncytial virus (RSV), rhinovirus, and seasonal coronaviruses. Testing procedures and data coverage for included centers are presented in Supplementary Table 1. Due to a lack of data coverage, patients hospitalized at Pomeranian Medical University (PUM) in Poland were excluded from the analyses of UAT, and those hospitalized at Azienda Socio-Sanitaria Territoriale (ASST) in Italy were excluded from the analyses of respiratory virus testing. Classification of significant pathogens was based on our previous definition or, if not covered by this definition, performed by senior infectious disease consultants [2]. Only Karolinska Institutet (KI) in Sweden had access to quantitative cutoffs for LRT cultures, and the numbers of positive cultures meeting these cutoffs were assessed separately [2]. Classification of each organism per test modality is presented in Supplementary Table 2.

Other variables included age, sex, comorbidities, and COVID-19 vaccination status before the hospitalization, as previously described [13]. Data were also collected on hospital length of stay, intensive care unit admission and length of stay, and in-hospital mortality.

### Statistical Methods

First, we described characteristics of the study population by SARS-CoV-2 variant and coinfection status. Continuous variables were calculated as median (IQR) and categorical variables as frequencies (percentage). Characteristics of patients from the different centers were also described and compared with Kruskal-Wallis tests or χ^2^ tests.

Second, we determined the proportion of patients in each variant group who had each microbiological test modality performed and their corresponding positivity rate. These data were also calculated for each center to assess whether testing frequencies and positivity rates differed across centers. Identified pathogens were presented overall and per SARS-CoV-2 variant group. Pathogens for patients hospitalized at KI with COVID-19 as the main diagnosis were identified to understand which ones occurred in this patient population, which was likely admitted *due* to COVID-19 rather than simply with it.

The occurrence of coinfections was presented overall and per SARS-CoV-2 variant. Unadjusted and adjusted risk ratios (RRs) with corresponding 95% CIs were determined for having a coinfection among patients with an Omicron infection as compared with preceding variants (categorized as pre-Omicron). All models were defined a priori according to previous literature as well as subject matter knowledge within the EuCARE consortium. RRs were obtained from modified Poisson regression models with 95% CIs based on the robust sandwich variance estimator. These analyses were also performed for 11 subgroups: tested patients only (ie, any of the test modalities performed), KI only, KI only with COVID-19 as main diagnosis, all other centers, patients aged <70 years, patients aged ≥70 years, patients who were immunocompromised, patients who were not immunocompromised, patients who were unvaccinated, patients who were vaccinated, and bacterial coinfections only. Adjusted models included age, sex, center, all investigated comorbidities, number of comorbidities, COVID-19 vaccination status, and month of hospital admission (January–December). Admission month was included to elucidate a potential confounding effect of seasonality, such as differences in circulating pathogens in the community [14, 15]. Adjusted models were modified to fit each subgroup analysis.

Unadjusted and adjusted modified Poisson regression models were used to understand factors associated with having a coinfection. Associations between age, sex, comorbidities, and COVID-19 vaccination status and having a coinfection were analyzed. For comorbidities and COVID-19 vaccination status, adjusted models included age, sex, and center.

The effect of coinfections on in-hospital mortality was investigated. This was done overall and for the subgroups, as well as for patients with pre-Omicron and Omicron infections. RRs with corresponding 95% CIs for in-hospital mortality in patients with and without a coinfection were calculated by modified Poisson regression models. Adjusted models included age, sex, center, comorbidities, number of comorbidities, COVID-19 vaccination status, SARS-CoV-2 variant, and calendar time. Calendar time was modeled as restricted cubic splines with knots set at 1 July 2020, 1 October 2021, and 9 March 2022, as previously described [10]. As a sensitivity analysis, 28-day in-hospital mortality by coinfection status was modeled with Fine-Gray subdistribution hazard models with alive discharge as a competing event, as previously described [13].

Data on COVID-19 vaccination status and intensive care unit admission were missing for 473 and 294 patients, respectively. The 473 patients without available COVID-19 vaccination status were excluded from regression models including this variable. An alpha level of .05 was used, and all analyses were performed in R version 4.4.1.

## RESULTS

A total of 29 564 patients were classified as having a wild type (n = 12 601), Alpha (n = 5256), Delta (n = 2433), or Omicron (n = 9274) infection (Supplementary Figure 2). The percentage of patients based on a sequenced sample was 4% (n = 563) for wild type, 17% (n = 918) for Alpha, 39% (n = 937) for Delta, and 31% (n = 2897) for Omicron. The number of patients from each country was as follows: 1692, Germany (Heinrich-Heine University); 2501, Italy (ASST); 1167, Lithuania (Vilnius University Hospital Santaros Klinikos [VULSK]); 2618, Poland (PUM); and 21 586, Sweden (KI).

### Characteristics of Patients With and Without Coinfections

Patients with a verified coinfection were older than patients without a verified coinfection in the wild type and Alpha groups (Table 1). A majority of patients were male for all 4 variants. Most comorbidities were more prevalent among patients with coinfections than patients without coinfections. In-hospital mortality rates were higher for individuals with coinfection vs without for all SARS-CoV-2 variant groups. The percentage of patients aged ≥70 years was >30% for all centers except for VULSK (14%, 162/1167; Supplementary Table 3). The percentage of patients having an Omicron infection was 26% (655/2501) for ASST, 40% (680/1692) for Heinrich-Heine University, 36% (7818/21 586) for KI, 1% (38/2618) for PUM, and 7% (83/1167) for VULSK. In-hospital mortality ranged from 5% (59/1167) in VULSK to 21% (531/2501) in ASST.

**Table 1.** Characteristics of the Study Population by SARS-CoV-2 Variant Group and Verified Coinfection

	Wild Type (n = 12 601)		Alpha (n = 5256)		Delta (n = 2433)		Omicron (n = 9274)	
Variable	No Coinfection (n = 12 274)	Coinfection (n = 327)	No Coinfection (n = 5151)	Coinfection (n = 105)	No Coinfection (n = 2356)	Coinfection (n = 77)	No Coinfection (n = 8537)	Coinfection (n = 737)
Age, y	66.0 (54.0–78.0)	75.0 (60.0–84.0)	61.0 (49.0–72.0)	67.0 (55.0–76.0)	59.0 (44.0–73.0)	59.0 (51.0–76.0)	75.0 (60.0–84.0)	75.0 (63.0–83.0)
Age category, y								
18–39	998 (8.1)	20 (6.1)	543 (10.5)	7 (6.7)	429 (18.2)	11 (14.3)	831 (9.7)	40 (5.4)
40–49	1296 (10.6)	32 (9.8)	761 (14.8)	10 (9.5)	391 (16.6)	6 (7.8)	540 (6.3)	33 (4.5)
50–59	2238 (18.2)	28 (8.6)	1115 (21.6)	17 (16.2)	373 (15.8)	22 (28.6)	748 (8.8)	64 (8.7)
60–69	2502 (20.4)	47 (14.4)	1216 (23.6)	23 (21.9)	439 (18.6)	7 (9.1)	1155 (13.5)	136 (18.5)
70–79	2488 (20.3)	85 (26.0)	936 (18.2)	30 (28.6)	364 (15.4)	16 (20.8)	2087 (24.4)	204 (27.7)
≥80	2752 (22.4)	115 (35.2)	580 (11.3)	18 (17.1)	360 (15.3)	15 (19.5)	3176 (37.2)	260 (35.3)
Male sex	7369 (60.0)	202 (61.8)	3070 (59.6)	65 (61.9)	1318 (55.9)	39 (50.6)	4525 (53.0)	407 (55.2)
Comorbidities								
Cancer	782 (6.4)	37 (11.3)	252 (4.9)	9 (8.6)	145 (6.2)	8 (10.4)	1134 (13.3)	111 (15.1)
Cardiac or cerebrovascular disease	3304 (26.9)	131 (40.1)	927 (18.0)	29 (27.6)	471 (20.0)	30 (39.0)	3488 (40.9)	326 (44.2)
Chronic kidney disease	1270 (10.3)	56 (17.1)	341 (6.6)	15 (14.3)	251 (10.7)	16 (20.8)	1561 (18.3)	156 (21.2)
Chronic liver disease	305 (2.5)	15 (4.6)	85 (1.7)	5 (4.8)	55 (2.3)	8 (10.4)	278 (3.3)	39 (5.3)
Chronic lung disease	1888 (15.4)	86 (26.3)	638 (12.4)	22 (21.0)	237 (10.1)	22 (28.6)	1782 (20.9)	224 (30.4)
Diabetes	2753 (22.4)	97 (29.7)	851 (16.5)	33 (31.4)	414 (17.6)	14 (18.2)	1972 (23.1)	194 (26.3)
Hypertension	5391 (43.9)	184 (56.3)	1784 (34.6)	50 (47.6)	794 (33.7)	32 (41.6)	4840 (56.7)	432 (58.6)
Immunocompromised	983 (8.0)	47 (14.4)	376 (7.3)	15 (14.3)	188 (8.0)	11 (14.3)	1577 (18.5)	161 (21.8)
Neurologic conditions	989 (8.1)	54 (16.5)	162 (3.1)	8 (7.6)	135 (5.7)	8 (10.4)	1147 (13.4)	100 (13.6)
Obesity	2421 (19.7)	66 (20.2)	1161 (22.5)	25 (23.8)	365 (15.5)	16 (20.8)	1353 (15.8)	123 (16.7)
No. of comorbidities								
0	3552 (28.9)	41 (12.5)	1946 (37.8)	22 (21.0)	943 (40.0)	14 (18.2)	1411 (16.5)	83 (11.3)
1	2961 (24.1)	71 (21.7)	1382 (26.8)	21 (20.0)	558 (23.7)	18 (23.4)	1640 (19.2)	137 (18.6)
2	2460 (20.0)	63 (19.3)	872 (16.9)	28 (26.7)	384 (16.3)	16 (20.8)	1864 (21.8)	150 (20.4)
3	1756 (14.3)	79 (24.2)	538 (10.4)	13 (12.4)	261 (11.1)	11 (14.3)	1735 (20.3)	157 (21.3)
≥4	1545 (12.6)	73 (22.3)	413 (8.0)	21 (20.0)	210 (8.9)	18 (23.4)	1887 (22.1)	210 (28.5)
COVID-19 vaccination doses^a^								
Unvaccinated	12 157 (99.9)	322 (100.0)	4622 (93.2)	78 (83.0)	1522 (66.2)	39 (52.0))	1591 (18.8)	97 (13.3)
1 dose	13 (0.1)	0 (0.0)	257 (5.2)	12 (12.8)	166 (7.2)	5 (6.7)	162 (1.9)	18 (2.5)
2 doses	0 (0.0)	0 (0.0)	79 (1.6)	4 (4.3)	554 (24.1)	31 (41.3)	1452 (17.2)	93 (12.7)
≥3 doses	0 (0.0)	0 (0.0)	0 (0.0)	0 (0.0)	56 (2.4)	0 (0.0)	5237 (62.0)	524 (71.6)
Hospital length of stay, d	7.0 (3.0–13.0)	8.0 (5.0–16.0)	7.0 (3.0–12.0)	8.0 (4.0–17.0)	8.0 (4.0–13.0)	9.0 (6.0–19.0)	4.0 (2.0–8.0)	6.0 (4.0–10.0)
Intensive care unit^b^								
Admission	1413 (11.5)	68 (20.9)	551 (10.7)	33 (31.4)	283 (12.1)	21 (27.3)	389 (4.7)	62 (8.6)
Length of stay, d	6.0 (2.0–13.0)	3.5 (1.0–8.3)	4.0 (2.0–10.0)	3.0 (1.0–11.0)	5.0 (2.0–12.0)	6.0 (0.0–11.0)	1.0 (1.0–4.0)	3.0 (1.0–5.8)
In-hospital mortality	1743 (14.2)	97 (29.7)	485 (9.4)	28 (26.7)	236 (10.0)	12 (15.6)	578 (6.8)	95 (12.9)

Data are presented as median (IQR) or No. (%).

^a^473 patients were excluded due to missing data.

^b^294 patients were excluded due to missing data.

### Testing Frequencies and Positivity Rates

**Figure 1.**
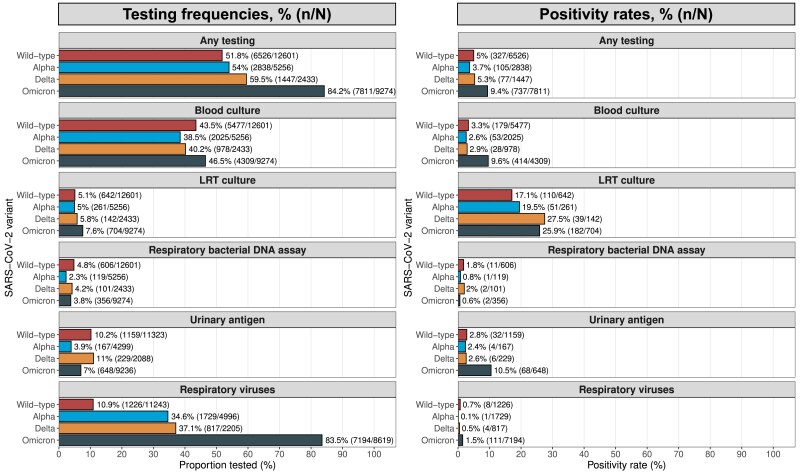
Testing frequencies and positivity rates per test modality and SARS-CoV-2 variant. Pomeranian Medical University was excluded from the analyses of urinary antigen testing due to lack of coverage in the data sources. Azienda Socio-Sanitaria Territoriale was excluded from the analyses of respiratory viruses due to lack of coverage in the data sources. Abbreviation: LRT, lower respiratory tract.

**Figure 2.**
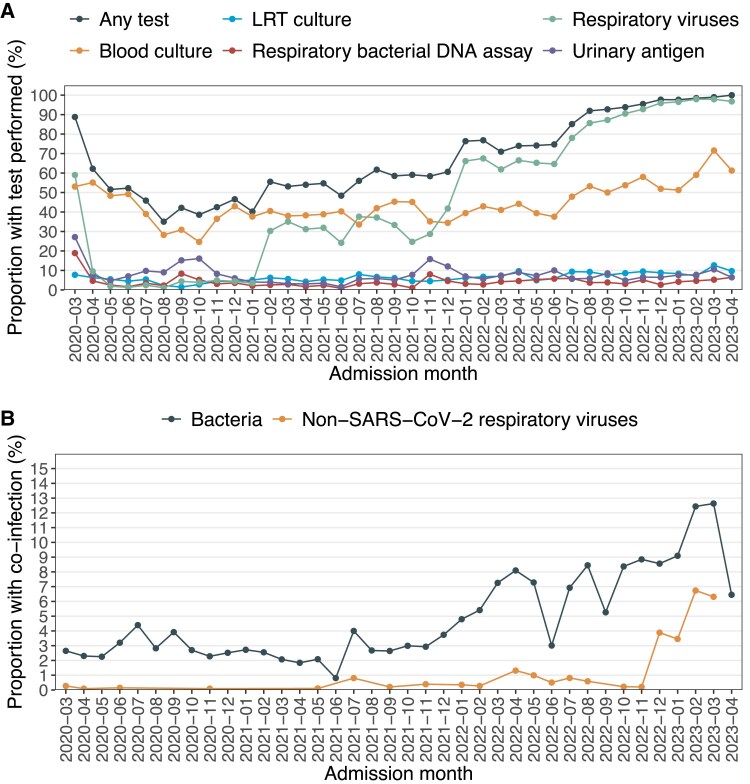
Testing frequencies (*A*) and occurrence of bacterial and viral coinfections (*B*) by calendar month of hospital admission.

Overall, the testing frequency was 63% (18 622/29 564) for any test, 43% (12 789/29 564) for blood cultures, 6% (1749/29 564) for LRT cultures, 4% (1182/29 564) for respiratory bacterial DNA assays, 8% (2203/26 946) for UAT, and 41% (10 966/27 063) for non–SARS-CoV-2 respiratory viruses. Testing frequencies were rather similar across the SARS-CoV-2 variants, except for respiratory viruses where patients in the Omicron group were tested more frequently (84%, 7194/8619) as compared with other variants (<40% for all variants; Figure 1 and Figure 2*A*). Testing frequencies varied across the centers (Supplementary Table 1, Supplementary Figure 3).

The positivity rate was 5% (674/12 789) for blood cultures, 22% (382/1749) for LRT cultures, 1% (16/1182) for respiratory bacterial DNA assays, 5% (110/2203) for UAT, and 1% (124/10 966) for non–SARS-CoV-2 respiratory viruses. The positivity rate for any testing (ie, number of patients with a detected pathogen by number of patients with any test) was 5.0% (327/6526) for wild type, 3.7% (105/2838) for Alpha, 5.3% (77/1447) for Delta, and 9.4% (737/7811) for Omicron. LRT cultures had the highest positivity rates across all SARS-CoV-2 variants. Of the 341 patients from KI with a positive LRT culture result, 86% (n = 292) had a significant number of colony-forming units per milliliter. The positivity rate for respiratory viruses was <2% for all variants. Positivity rates also differed across the centers (Supplementary Figure 3). Overall, the positivity rate was very low for VULSK (<0.5% for all test modalities).

### Identified Pathogens

A total of 93 pathogens were identified among 1246 patients (Supplementary Table 2). The most common pathogens were *Staphylococcus aureus* (n = 221 patients), *Escherichia coli* (n = 209), *Streptococcus pneumoniae* (n = 193), *Haemophilus influenzae* (n = 106), and *Klebsiella pneumoniae* (n = 68). When the analyses were restricted to patients from KI with a main diagnosis of COVID-19, the most common pathogens were *S aureus* (n = 113), *S pneumoniae* (n = 81), *H influenzae* (n = 70), *E coli* (n = 36), and *Moraxella catarrhalis* (n = 32).

A total of 124 patients had a coinfection caused by a non–SARS-CoV-2 respiratory virus. The most commonly identified viruses were influenza A (n = 51 patients), RSV (n = 43), and rhinovirus (n = 10). Among patients who had a respiratory virus coinfection, 90% (111/124) were in the Omicron group.

*S aureus* was the most common pathogen among patients in the wild type (n = 87) and Alpha (n = 31) groups, as compared with *S pneumoniae* (n = 16) in the Delta group and *E coli* (n = 141) in the Omicron group (Supplementary Table 4). When the analyses were restricted to patients from KI with a main diagnosis of COVID-19, *S pneumoniae* (n = 42), *H influenzae* (n = 33), and *S aureus* (n = 28) were the most commonly identified pathogens among patients in the Omicron group (Supplementary Table 5). Of patients with a bacterial pathogen, 59% (674/1140) had a positive blood culture result, 33% (382/1140) had a positive LRT culture result, 1.4% (16/1140) had a positive respiratory bacterial DNA assay result, and 9.6% (110/1140) had a positive UAT result. Corresponding numbers for the Omicron group were 64.4% (414/643), 28.3% (182/643), 0.3% (2/643), and 10.6% (68/643).

### Occurrence and Risk of Coinfections by SARS-CoV-2 Variant

The occurrence of any coinfection was 2.6% (327/12 601) for wild type, 2.0% (105/5256) for Alpha, 3.2% (77/2433) for Delta, and 7.9% (737/9274) for Omicron. When the analyses were restricted to individuals who had at least 1 test modality performed, the occurrence was 5.0% (327/6526) for wild type, 3.7% (105/2838) for Alpha, 5.3% (77/1447) for Delta, and 9.4% (737/7811) for Omicron. The proportion with a bacterial and viral coinfection per calendar month of hospital admission is presented in Figure 2*B*. While bacterial coinfections increased after Omicron emerged (December 2021), non–SARS-CoV-2 respiratory viruses did not increase substantially until the 2022–2023 winter season.

When compared with pre-Omicron, unadjusted RRs for coinfection were significantly increased for Omicron overall and for all subgroup analyses (Table 2). The adjusted RR (95% CI) for coinfection in the entire study population was 1.88 (1.53–2.32) for Omicron when compared with pre-Omicron. Large differences were observed in RRs for patients aged <70 vs ≥70 years, with stronger associations observed for patients <70 years old. When the analyses were restricted to bacterial coinfections, the adjusted RR (95% CI) was 1.74 (1.40–2.16) for Omicron as compared with pre-Omicron.

**Table 2.** Occurrence and Risk of Coinfection for Omicron vs Pre-Omicron for the Overall Study Population and Predefined Subgroups

		Risk Ratio (95% CI)	
Group: SARS-CoV-2 Variant	Occurrence, % (No.)	Unadjusted	Adjusted^a^
Overall			
Pre-Omicron	2.5 (509/20 290)	1.0 [Reference]	1.0 [Reference]^b^
Omicron	7.9 (737/9274)	3.17 (2.84–3.54)	1.88 (1.53–2.32)
Tested patients			
Pre-Omicron	4.7 (509/10 811)	1.0 [Reference]	1.0 [Reference]^b^
Omicron	9.4 (737/7811)	2.00 (1.80–2.24)	1.50 (1.23–1.82)
KI only			
Pre-Omicron	2.8 (388/13 768)	1.0 [Reference]	1.0 [Reference]^c^
Omicron	8.8 (687/7818)	3.12 (2.76–3.52)	1.99 (1.56–2.54)
KI only, COVID-19 main diagnosis			
Pre-Omicron	2.3 (277/11 846)	1.0 [Reference]	1.0 [Reference]^c^
Omicron	5.8 (191/3294)	2.48 (2.07–2.97)	1.50 (1.07–2.11)
All centers except KI			
Pre-Omicron	1.9 (121/6522)	1.0 [Reference]	1.0 [Reference]^d^
Omicron	3.4 (50/1456)	1.85 (1.34–2.56)	1.48 (.85–2.57)
Age <70 y			
Pre-Omicron	1.8 (230/12 531)	1.0 [Reference]	1.0 [Reference]^e^
Omicron	7.7 (273/3547)	4.19 (3.53–4.98)	2.84 (2.06–3.92)
Age ≥70 y			
Pre-Omicron	3.6 (279/7759)	1.0 [Reference]	1.0 [Reference]^f^
Omicron	8.1 (464/5727)	2.25 (1.95–2.60)	1.33 (1.01–1.74)
Immunocompromised			
Pre-Omicron	4.5 (73/1620)	1.0 [Reference]	1.0 [Reference]^g^
Omicron	9.3 (161/1738)	2.06 (1.57–2.69)	1.76 (1.04–2.99)
Not immunocompromised			
Pre-Omicron	2.3 (436/18 670)	1.0 [Reference]	1.0 [Reference]^g^
Omicron	7.6 (576/7536)	3.27 (2.90–3.70)	1.86 (1.48–2.34)
Unvaccinated			
Pre-Omicron	2.3 (439/18 740)	1.0 [Reference]	1.0 [Reference]^h^
Omicron	5.7 (97/1688)	2.45 (1.98–3.04)	1.77 (1.35–2.31)
Vaccinated			
Pre-Omicron	4.4 (52/1177)	1.0 [Reference]	1.0 [Reference]^h^
Omicron	8.5 (635/7486)	1.92 (1.46–2.53)	1.60 (1.20–2.14)
Bacterial coinfections only			
Pre-Omicron	2.4 (497/20 290)	1.0 [Reference]	1.0 [Reference]^b^
Omicron	6.9 (643/9274)	2.83 (2.52–3.17)	1.74 (1.40–2.16)

Abbreviations: ASST, Azienda Socio-Sanitaria Territoriale; HHU, Heinrich-Heine University; KI, Karolinska Institutet; PUM, Pomeranian Medical University; VULSK, Vilnius University Hospital Santaros Klinikos.

^a^473 patients were excluded from this model due to missing data on COVID-19 vaccination.

^b^Adjusted for age category (18–39, 40–49, 50–59, 60–69, 70–79, ≥80 years), sex (male, female), center (ASST, HHU, KI, PUM, VULSK), all comorbidities considered in the study (Table 1), number of comorbidities (0, 1, 2, 3, ≥4), COVID-19 vaccination status (unvaccinated, 1 dose, 2 doses, ≥3 doses), and month (January–December).

^c^Adjusted for age category (18–39, 40–49, 50–59, 60–69, 70–79, ≥80 years), sex (male, female), all comorbidities considered in the study (Table 1), number of comorbidities (0, 1, 2, 3, ≥4), COVID-19 vaccination status (unvaccinated, 1 dose, 2 doses, ≥3 doses), and month (January–December).

^d^Adjusted for age category (18–39, 40–49, 50–59, 60–69, 70–79, ≥80 years), sex (male, female), center (ASST, HHU, PUM, VULSK), all comorbidities considered in the study (Table 1), number of comorbidities (0, 1, 2, 3, ≥4), COVID-19 vaccination status (unvaccinated, 1 dose, 2 doses, ≥3 doses), and month (January–December).

^e^Adjusted for age category (18–39, 40–49, 50–59, 60–69 years), sex (male, female), center (ASST, HHU, KI, PUM, VULSK), all comorbidities considered in the study (Table 1), number of comorbidities (0, 1, 2, 3, ≥4), COVID-19 vaccination status (unvaccinated, 1 dose, 2 doses, ≥3 doses), and month (January–December).

^f^Adjusted for age category (70–79, ≥80 years), sex (male, female), center (ASST, HHU, KI, PUM, VULSK), all comorbidities considered in the study (Table 1), number of comorbidities (0, 1, 2, 3, ≥4), COVID-19 vaccination status (unvaccinated, 1 dose, 2 doses, ≥3 doses), and month (January–December).

^g^Adjusted for age category (18–39, 40–49, 50–59, 60–69, 70–79, ≥80 years), sex (male, female), center (ASST, HHU, KI, PUM, VULSK), all comorbidities considered in the study except immunocompromised state (Table 1), number of comorbidities (0, 1, 2, 3, ≥4), COVID-19 vaccination status (unvaccinated, 1 dose, 2 doses, ≥3 doses), and month (January–December).

^h^Adjusted for age category (18–39, 40–49, 50–59, 60–69, 70–79, ≥80 years), sex (male, female), center (ASST, HHU, KI, PUM, VULSK), all comorbidities considered in the study (Table 1), number of comorbidities (0, 1, 2, 3, ≥4), and month (January–December).

### Factors Associated With Coinfections

**Figure 3.**
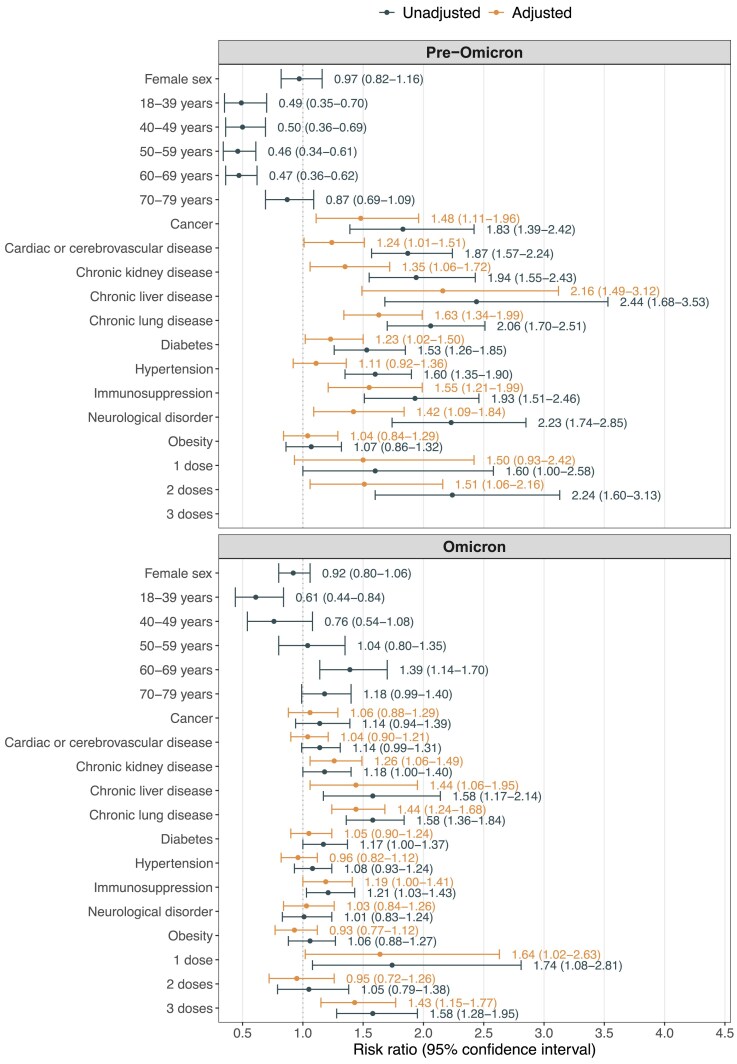
Factors associated with a verified coinfection during pre-Omicron and Omicron periods. The adjusted risk ratios (95% CIs) were adjusted for age group (18–39, 40–49, 50–59, 60–69, 70–79, ≥80 years), sex (male, female), and center (Azienda Socio-Sanitaria Territoriale, Heinrich-Heine University, Karolinska Institutet, Pomeranian Medical University, Vilnius University Hospital Santaros Klinikos).

Stronger associations were observed for age and the studied comorbidities in the pre-Omicron group vs the Omicron group (Figure 3). In the Omicron group, chronic lung and liver diseases were the comorbidities with the strongest association with having a verified coinfection (adjusted RR [95% CI]: chronic liver disease, 1.44 [1.06–1.95]; chronic lung disease, 1.44 [1.24–1.68]). When compared with unvaccinated patients in the Omicron group, patients who received 3 doses of COVID-19 vaccine had an increased risk of a verified coinfection (1.43 [1.15–1.77]).

### Association of Coinfections With In-hospital Mortality

The in-hospital mortality rate was 19% (232/1246) for patients with a coinfection and 11% (3042/28 318) for patients without a coinfection (Table 3). The adjusted RR (95% CI) for in-hospital mortality was 1.69 (1.49–1.91) for patients with vs without a coinfection. The RR (95% CI) was 1.58 (1.36–1.84) for patients in the pre-Omicron group and 1.87 (1.52–2.30) in the Omicron group. Among unvaccinated patients, in-hospital mortality was 25% (132/536) for patients with a coinfection and 12% (2378/19 892) for patients without a coinfection. The adjusted RR (95% CI) for in-hospital mortality was 1.72 (1.52–1.95) for patients with vs without a bacterial coinfection. Similar associations and trends were observed when in-hospital mortality was modeled with Fine-Gray subdistribution hazard models (Supplementary Table 6). The identified pathogens among the patients dying in-hospital with a verified coinfection overall, as well as for the Omicron group, are presented in Supplementary Table 7. The most common pathogens overall and in the Omicron group among patients who died were *S aureus*, *S pneumoniae*, *E coli*, and *K pneumoniae*.

**Table 3.** Occurrence and Risk of In-hospital Mortality by Coinfection Status for the Overall Study Population and Predefined Subgroups

		Risk Ratio (95% CI)	
Group: Coinfection	In-hospital Mortality, % (No.)	Unadjusted	Adjusted^a^
Overall			
No	10.7 (3042/28 318)	1 [Reference]	1 [Reference]^b^
Yes	18.6 (232/1246)	1.73 (1.54–1.96)	1.69 (1.49–1.91)
Pre-Omicron			
No	12.5 (2464/19 781)	1 [Reference]	1 [Reference]^c^
Yes	26.9 (137/509)	2.16 (1.86–2.51)	1.58 (1.36–1.84)
Omicron			
No	6.8 (578/8537)	1 [Reference]	1 [Reference]^d^
Yes	12.9 (95/737)	1.90 (1.55–2.33)	1.87 (1.52–2.30)
Tested patients			
No	11.4 (1981/17 376)	1 [Reference]	1 [Reference]^b^
Yes	18.6 (232/1246)	1.63 (1.44–1.85)	1.53 (1.35–1.73)
KI only			
No	9.2 (1886/20 511)	1 [Reference]	1 [Reference]^e^
Yes	16.5 (177/1075)	1.79 (1.55–2.06)	1.71 (1.49–1.97)
KI only, COVID-19 main diagnosis			
No	10.5 (1545/14 672)	1 [Reference]	1 [Reference]^e^
Yes	19.2 (90/468)	1.83 (1.51–2.21)	1.53 (1.26–1.85)
All centers except KI			
No	14.8 (1156/7807)	1 [Reference]	1 [Reference]^f^
Yes	32.2 (55/171)	2.17 (1.74–2.72)	1.51 (1.18–1.95)
Age <70 y			
No	4.5 (706/15 575)	1 [Reference]	1 [Reference]^g^
Yes	11.9 (60/503)	2.63 (2.05–3.37)	2.52 (1.94–3.27)
Age ≥70 y			
No	18.3 (2336/12 743)	1 [Reference]	1 [Reference]^h^
Yes	23.1 (172/743)	1.26 (1.10–1.45)	1.52 (1.33–1.75)
Immunocompromised			
No	11.5 (358/3124)	1 [Reference]	1 [Reference]^i^
Yes	12.4 (29/234)	1.08 (.76–1.54)	1.07 (.73–1.56)
Not immunocompromised			
No	10.7 (2684/25 194)	1 [Reference]	1 [Reference]^i^
Yes	20.1 (203/1012)	1.88 (1.66–2.14)	1.83 (1.61–2.08)
Unvaccinated			
No	12.0 (2378/19 892)	1 [Reference]	1 [Reference]^j^
Yes	24.6 (132/536)	2.06 (1.77–2.40)	1.62 (1.39–1.89)
Vaccinated			
No	7.6 (604/7976)	1 [Reference]	1 [Reference]^j^
Yes	12.8 (88/687)	1.69 (1.37–2.09)	1.73 (1.40–2.14)
Bacterial coinfections only			
No	10.7 (3050/28 424)	1 [Reference]	1 [Reference]^b^
Yes	19.6 (224/1140)	1.83 (1.62–2.07)	1.72 (1.52–1.95)

Abbreviations: ASST, Azienda Socio-Sanitaria Territoriale; HHU, Heinrich-Heine University; KI, Karolinska Institutet; PUM, Pomeranian Medical University; VULSK, Vilnius University Hospital Santaros Klinikos.

^a^473 patients were excluded from this model due to missing data on COVID-19 vaccination.

^b^Adjusted for age category (18–39, 40–49, 50–59, 60–69, 70–79, ≥80 years), sex (male, female), center (ASST, HHU, KI, PUM, VULSK), all comorbidities considered in the study (Table 1), number of comorbidities (0, 1, 2, 3, ≥4), COVID-19 vaccination status (unvaccinated, 1 dose, 2 doses, ≥3 doses), SARS-CoV-2 variant (wild type, Alpha, Delta, Omicron), and calendar time.

^c^Adjusted for age category (18–39, 40–49, 50–59, 60–69, 70–79, ≥80 years), sex (male, female), center (ASST, HHU, KI, PUM, VULSK), all comorbidities considered in the study (Table 1), number of comorbidities (0, 1, 2, 3, ≥4), COVID-19 vaccination status (unvaccinated, 1 dose, 2 doses, ≥3 doses), SARS-CoV-2 variant (wild type, Alpha, Delta), and calendar time.

^d^Adjusted for age category (18–39, 40–49, 50–59, 60–69, 70–79, ≥80 years), sex (male, female), center (ASST, HHU, KI, PUM, VULSK), all comorbidities considered in the study (Table 1), number of comorbidities (0, 1, 2, 3, ≥4), COVID-19 vaccination status (unvaccinated, 1 dose, 2 doses, ≥3 doses), and calendar time.

^e^Adjusted for age category (18–39, 40–49, 50–59, 60–69, 70–79, ≥80 years), sex (male, female), all comorbidities considered in the study (Table 1), number of comorbidities (0, 1, 2, 3, ≥4), COVID-19 vaccination status (unvaccinated, 1 dose, 2 doses, ≥3 doses), SARS-CoV-2 variant (wild type, Alpha, Delta, Omicron), and calendar time.

^f^Adjusted for age category (18–39, 40–49, 50–59, 60–69, 70–79, ≥80 years), sex (male, female), center (ASST, HHU, PUM, VULSK), all comorbidities considered in the study (Table 1), number of comorbidities (0, 1, 2, 3, ≥4), COVID-19 vaccination status (unvaccinated, 1 dose, 2 doses, ≥3 doses), SARS-CoV-2 variant (wild type, Alpha, Delta, Omicron), and calendar time.

^g^Adjusted for age category (18–39, 40–49, 50–59, 60–69), sex (male, female), center (ASST, HHU, KI, PUM, VULSK), all comorbidities considered in the study (Table 1), number of comorbidities (0, 1, 2, 3, ≥4), COVID-19 vaccination status (unvaccinated, 1 dose, 2 doses, ≥3 doses), SARS-CoV-2 variant (wild type, Alpha, Delta, Omicron), and calendar time.

^h^Adjusted for age category (70–79, ≥80 years), sex (male, female), center (ASST, HHU, KI, PUM, VULSK), all comorbidities considered in the study (Table 1), number of comorbidities (0, 1, 2, 3, ≥4), COVID-19 vaccination status (unvaccinated, 1 dose, 2 doses, ≥3 doses), SARS-CoV-2 variant (wild type, Alpha, Delta, Omicron), and calendar time.

^i^Adjusted for age category (18–39, 40–49, 50–59, 60–69, 70–79, ≥80 years), sex (male, female), center (ASST, HHU, KI, PUM, VULSK), all comorbidities considered in the study except immunocompromised state (Table 1), number of comorbidities (0, 1, 2, 3, ≥4), COVID-19 vaccination status (unvaccinated, 1 dose, 2 doses, ≥3 doses), SARS-CoV-2 variant (wild type, Alpha, Delta, Omicron), and calendar time.

^j^Adjusted for age category (18–39, 40–49, 50–59, 60–69, 70–79, ≥80 years), sex (male, female), center (ASST, HHU, KI, PUM, VULSK), all comorbidities considered in the study (Table 1), number of comorbidities (0, 1, 2, 3, ≥4), SARS-CoV-2 variant (wild type, Alpha, Delta, Omicron), and calendar time.

## DISCUSSION

In this multinational study, we found an increased occurrence and risk of coinfections in patients hospitalized during the Omicron period as compared with preceding variants. The occurrence of coinfections with respiratory viruses was low (≤1.5% for all variants) despite extensive testing, although these increased during the 2022–2023 season. Across countries, testing practices differed, but coinfection was low. Adjusted RRs for coinfection were significantly increased for patients hospitalized with Omicron as compared with preceding variants. These results were consistent across most subgroup analyses. Furthermore, having a coinfection was associated with an increased risk of in-hospital mortality.

We observed an overall occurrence of coinfections of 4.2%, which is in line with findings from previous studies [1–3]. In addition, we found a low prevalence of viral coinfections, which is in line with results from a meta-analysis, finding a 2% pooled prevalence of coinfection with influenza virus [11]. Mechanistic reasons for this are poorly understood, with conflicting results from in vitro studies and clinical studies [16, 17]. Studies restricted to hospitalized patients ought not to be used for elucidating viral interference [18]. A study on human airway epithelium infected with SARS-CoV-2 and influenza demonstrated that influenza A interfered with SARS-CoV-2 viruses, but the opposite interference was not observed [19]. Since patients are often sampled for viruses by multiplex assays, the possibility to detect virus-virus interactions has increased. Despite a large proportion of the study population being sampled for at least 1 non–SARS-CoV-2 respiratory virus, <0.5% had a verified viral coinfection. However, an increased occurrence of respiratory virus coinfections was observed during the 2022–2023 season, with >4% of patients coinfected from December 2022 until April 2023. The increasing but still rather low occurrence of viral coinfections observed during the Omicron period might be due to differential testing strategies throughout the periods of the pandemic.

A Swedish study demonstrated an occurrence of a bacterial coinfection of 4% in patients with COVID-19, as opposed to 27% and 29% for influenza and RSV, respectively [2]. What remains to be understood is whether the large differences in bacterial coinfections are driven by viral mechanisms and/or differences in severity of virus infection, requiring further coinfections to lead to hospitalization events. For SARS-CoV-2, the latter is supported by the finding that patients with previous immunity (vaccination) toward SARS-CoV-2 had an increased risk of coinfections. Data on coinfections in patients with Omicron infections remain rather scarce but corroborate our finding of an increased occurrence since Omicron emerged [5–7]. Bacterial coinfections were detected in 6.9% during the Omicron period, which was somewhat higher than that during the early phases of the pandemic. Our results indicate that should empiric antibiotics be administered due to suspicion of bacterial coinfections, they should be targeted against *S aureus*, *E coli*, *K pneumoniae*, *S pneumoniae*, and *H influenzae*.

Strengths of our study include the same inclusion criteria used across all countries from North, Central, South, and East Europe. Detailed microbiological data were available for several test modalities, with classifications of significance done by infectious disease consultants, from a period >3 years since SARS-CoV-2 emerged. Data were available for important potential confounders, such as age, sex, comorbidities, and COVID-19 vaccination status. Finally, the large sample size enabled several subgroup analyses for the occurrence of coinfection and risk of in-hospital mortality.

Limitations include that 73% of the study population was from KI; thus, Swedish data had a strong impact on the results. This could be observed in the RRs excluding KI, where statistical precision decreased and no significant differences between Omicron and pre-Omicron were observed. Furthermore, PUM and VULSK had access to data on only a small number of patients hospitalized with Omicron. The substantial differences in testing practices across the centers might have influenced the observed occurrences of coinfections. However, it could also be seen as a strength to have data from countries with different clinical indications and testing routines. It is possible that patients presented with coinfections not covered by the test modalities, such as fungal infections. It is further plausible that some of the findings classified as significant pathogens were of limited clinical relevance and vice versa, where the focus of this study was on pathogens likely to have been acquired in a community setting. To ascertain whether a patient is hospitalized due to or with COVID-19 has become increasingly difficult, especially since the emergence of Omicron. It is plausible that a larger proportion of patients were hospitalized due to reasons other than COVID-19, such as coinfections and superinfections, for the Omicron group when compared with the other variants, possibly explaining the increased risk of coinfections observed. Yet, results were consistent when the analyses were restricted to patients with a main diagnosis of COVID-19 from KI. It could be argued that having access to data on these patients admitted primarily due to reasons other than COVID-19 can be a strength, as it possibly indicates that more than the SARS-CoV-2 infection is required for patients to require hospitalization, a phenomenon more similar to that for patients with respiratory virus infections such as influenza and RSV [2]. Having access to data on the need for oxygen support, such as noninvasive ventilation and mechanical ventilation, would have been useful to understand how the severity of COVID-19 differed during the variant periods. The percentage of patients based on a sequenced SARS-CoV-2 sample was low, as previously described [13]. Another limitation of our study is the increased probability of type 1 error due to multiple subgroup analyses. Finally, we were not able to take the previous infection into account, as described before [13].

## CONCLUSIONS

Bacterial and viral coinfections, while still less prevalent when compared with hospitalizations for influenza or RSV, increased since the emergence of Omicron. Furthermore, having a coinfection was associated with an increased risk of in-hospital mortality as compared with having no coinfection. These results call for vigilance among health care practitioners seeing patients hospitalized with COVID-19, with continued monitoring of coinfections in such patient populations.

## Supplementary Material

jiaf167_Supplementary_Data

## Supplementary Data

Supplementary materials are available at *The Journal of Infectious Diseases* online (http://jid.oxfordjournals.org/). Supplementary materials consist of data provided by the author that are published to benefit the reader. The posted materials are not copyedited. The contents of all supplementary data are the sole responsibility of the authors. Questions or messages regarding errors should be addressed to the author.

## Notes

***Author contributions.*** All authors were involved in conceptualization, data collection, investigation, methodology, and resources. Funding was acquired by A. S., F. I., and M. Z. F. I. and P. H. were responsible for the project administration. A. S., P. N., and B.-E. O. J. supervised the project. I. F. designed and created the EuCARE database. P. H. curated and visualized the data and wrote the original draft. P. H. had full access to all the data in the study, performed the formal analyses, and validated the results. All authors contributed to reviewing and editing the manuscript.

***Data availability*.** All code used in the study is available upon request by the corresponding author. The individual participant data underlying this article were subject to ethical approval and cannot be shared publicly. Data from the deidentified data sources are not freely available due to protection of the personal integrity of the participants.

***Ethics approval*.** The study (ClinicalTrials.gov NCT05463380) was approved by ethical review authorities for the participating centers: Comitato Etico Milano Area 1–Ospedale Luigi Sacco (approved 22 June 2022), Ethikkommission Medizinischen Fakultät der Heinrich-Heine-Universität Düsseldorf (approved 6 July 2022), Swedish Ethical Review Authority (approved 23 February 2022), Komisji Bioetycznej Pomorskiego Uniwersytetu Medycznego w Szczecinie (approved 28 September 2022), and Vilniaus regioninis biomedicininių tyrimų etikos komitetas (approved 29 April 2022).

***Financial support*.** This work was supported by the EuCARE Project, funded by the European Union's Horizon Europe Research and Innovation Programme (grant agreement 101046016).
