# Supplementary material for: Bacterial and Viral Coinfections in Adult Patients Hospitalized With COVID-19 Throughout the Pandemic: A Multinational Cohort Study in the EuCARE Project
Source: J Infect Dis. 2025 Apr 3;231(6):e1091–101. doi: 10.1093/infdis/jiaf167 (PMC12247819; doi:10.1093/infdis/jiaf167)
Supplement: jiaf167_Supplementary_Data [file jiaf167_supplementary_data.docx]

Supplementary material for

**Bacterial and viral co-infections in adult patients hospitalized with COVID-19 throughout the pandemic: A Multinational Cohort Study in the EuCARE Project**

**Authors:** Pontus Hedberg, Karol Serwin, Maria Francesca Greco, Joana P. V. Pereira, Dovile Juozapaite, Sara de Benedittis, Francesca Bai, Nadine Lübke, Tobias Wienemann, Iuri Fanti, Florian König, Nico Pfeifer, Rolf Kaiser, Maurizio Zazzi, Alessandro Cozzi-Lepri, Daniel Naumovas**,** Giulia Marchetti, Milosz Parczewski, Björn-Erik Ole Jensen, Francesca Incardona, Anders Sönnerborg, Pontus Nauclér

Correspondence to [pontus.hedberg@ki.se](mailto:pontus.hedberg@ki.se)

**Table of contents**

| **Content** | **Page** |
| --- | --- |
| Figure S1. Study overview | 3 |
| Table S1. Testing procedures and data sources/coverage for the centres | 4-8 |
| Table S2. Classification of contaminants and pathogens | 9-14 |
| Figure S2. Study flow chart | 15 |
| Table S3. Characteristics of the study population by centre | 16-17 |
| Figure S3. Testing frequencies and positivity rates per centre | 18 |
| Table S4. The most commonly identified organisms per SARS-CoV-2 variant | 19-20 |
| Table S5. The most commonly identified organisms per SARS-CoV-2 variant among patients from KI with a main diagnosis of COVID-19 | 21-22 |
| Table S6. Subdistribution hazard ratios for 28-day in-hospital mortality and risk ratios for in-hospital mortality by co-infection status for the overall study population as well as pre-defined subgroups | 23-26 |
| Table S7. Identified pathogens among patients dying in-hospital with a verified co-infection overall as well as during the Omicron period only | 27-28 |

**Figure S1. Study overview**

**
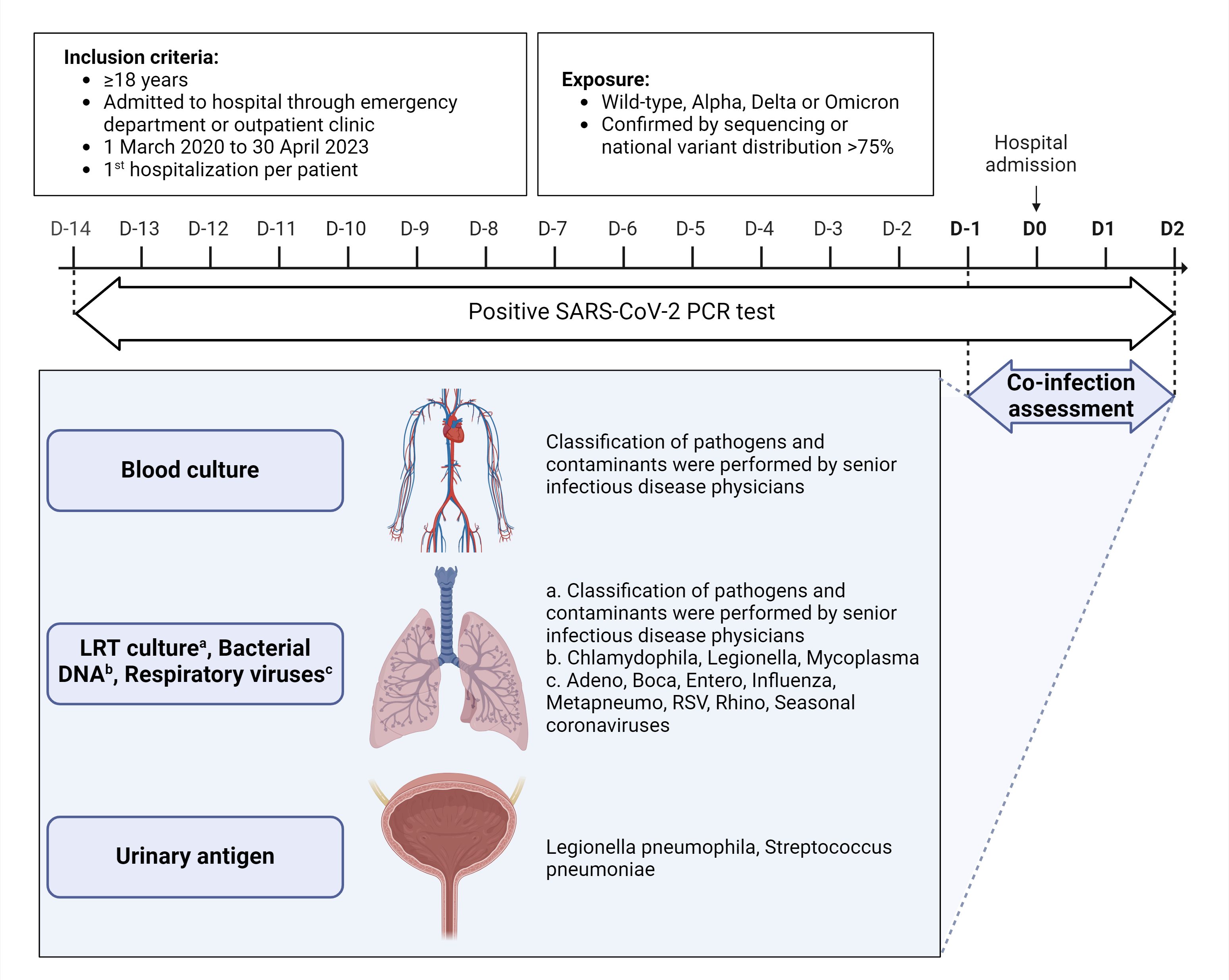
**

**Note:** Created in BioRender. Juozapaite, D. (2024) BioRender.com/k89r071

**Abbreviations:** D=Day, LRT=Lower respiratory tract, RSV=Respiratory syncytial virus, SARS-CoV-2=Severe acute respiratory syndrome coronavirus 2

**Table S1. Testing procedures and data sources/coverage for the centres**

| **Test modality** | **Center** | **Data sources/coverage** | **Indication/Testing procedure** |
| --- | --- | --- | --- |
| Blood culture | ASST | Data mostly ICU, Infectious Disease units, Pneumology units, General Medicine units, and Rehabilitation Wards. During the busiest period, medical and pulmonology departments would come and “specialize” in COVID-19 units, so a critical patient would be sent to those units. | Performed when the patient has fever, when fever >38°C a set of aereobic/anaerobic blood culture is performed. Usually at least 2 blood samples, drawn through a needle from different veins, are performed. |
| Blood culture | HHU | All units at the hospital | All pathogens were identified by MALDI-ToF using bioMérieux Vitek MS and antimicrobial susceptibility testing was performed using the bioMérieux Vitek II system, disk diffusion or gradient testing.  Blood cultures were processed using standard microbiological procedures using the bioMérieux VIRTUO™. Positive bottles were sub-cultured on standard agar for identification and susceptibility testing. |
| Blood culture | KI | All units at the hospital | Performed when the patient has fever and suspicion of systemic bacterial infection. |
| Blood culture | PUM | All units at the hospital | Aerobic, anaerobic, and fungal blood cultures are performed. These are not routinely performed in patients with COVID-19 unless there is a suspicion of a bacterial co-infection with a septic course. |
| Blood culture | VULSK | All units at the hospital | Performed on most patients, growing on different media for aerobes and anaerobes |
| LRT culture | ASST | Data mostly ICU, Infectious Disease units, Pneumology units, General Medicine units, and Rehabilitation Wards. During the busiest period, medical and pulmonology departments would come and “specialize” in COVID-19 units, so a critical patient would be sent to those units. | Performed when the patient has fever and symptoms of LRTI (cough or shortness of breath or dyspnea) and there is the suspicion of bacterial infection. |
| LRT culture | HHU | All units at the hospital | All pathogens were identified by MALDI-ToF using bioMérieux Vitek MS and antimicrobial susceptibility testing was performed using the bioMérieux Vitek II system, disk diffusion or gradient testing.  Lower respiratory tract specimens were inoculated onto chocolate agar, blood agar, and MacConkey agar and incubated at 37°C for 48h. One blood agar plate was incubated under anaerobic conditions, while the other plates were incubated in a 5% CO2. The plates were examined after 24 hours and again after 48 hours. |
| LRT culture | KI | All units at the hospital | Performed when the patient has fever and symptoms of LRTI and there is the suspicion of bacterial infection. |
| LRT culture | PUM | All units at the hospital | LRT cultures, particularly sputum cultures, are only performed if deemed crucial for diagnosing typical lower respiratory tract infections. Due to the lack of easy access to bronchoalveolar lavage (BAL), only sputum cultures are used for patients with a suspected bacterial superinfection. |
| LRT culture | VULSK | All units at the hospital | Performed only in severely ill patients |
| Respiratory bacterial DNA assay | ASST | Data mostly ICU, Infectious Disease units, Pneumology units, General Medicine units, and Rehabilitation Wards. During the busiest period, medical and pulmonology departments would come and “specialize” in COVID-19 units, so a critical patient would be sent to those units. | Performed on BAL, BAS and sputum with Kit Film Array Pneumonia Panel plus (Biomerieux) (from November 2022) |
| Respiratory bacterial DNA assay | HHU | All units at the hospital | The molecular genetic detection of Chlamydophila, Legionella pneumoniae and Mycoplasma pneumoniae from respiratory tract specimens was carried out using an in house multiplex-PCR. |
| Respiratory bacterial DNA assay | KI | All units at the hospital | Included in a comprehensive respiratory panel. Performed if suspicion of infection with atypical bacteria. |
| Respiratory bacterial DNA assay | PUM | All units at the hospital | Included in a comprehensive multiplex respiratory PCR panel from Qiastat Dx Analyzer (Qiagen, Germany) according to the manufacturer protocol. These assays are only routinely performed in patients with COVID-19 if a superinfection with another bacterial or viral agent is suspected. They are conducted sporadically in patients with confirmed COVID-19. |
| Respiratory bacterial DNA assay | VULSK | All units at the hospital | Not performed in COVID-19 patients |
| Urinary antigen | ASST | Data mostly ICU, Infectious Disease units, Pneumology units, General Medicine units, and Rehabilitation Wards. During the busiest period, medical and pulmonology departments would come and “specialize” in COVID-19 units, so a critical patient would be sent to those units. | Urinary antigen testing is for both S Penumoniae e L. pneumophila (immunochromatographic assay for the detection of both S. pneumoniae and L. pneumophila urinary antigen): Legionella Ag FIA (SD BIOSENSOR), S.pneumoniae Ag FIA (SD BIOSENSOR) (from March 2020) |
| Urinary antigen | HHU | All units at the hospital | No antigen test for S. pneumoniae from urine is carried out at the UKD. The detection of Legionella spp. antigen in urine is carried out using the BinaxNOW ™ Legionella Antigen card (Abbott, Cologne) |
| Urinary antigen | KI | All units at the hospital | Performed for Legionella pneumophila (serotype 1) and S. pneumoniae. Performed in patients with suspected bacterial pneumonia. |
| Urinary antigen | PUM | No access to this test modality | Performed for Legionella pneumophila (serotype 1) and S. pneumoniae. Ordered when severe bacterial infections are suspected. |
| Urinary antigen | VULSK | All units at the hospital | Performed only in severely ill patients |
| Respiratory viruses | ASST | No access to this test modality | Very limited respiratory viruses testing. In COVID-19 patients, these tests are not required unless there is a clinical worsening or a critical baseline situation. |
| Respiratory viruses | HHU | All units at the hospital | The detection of respiratory viruses took place when there was a clinical indication of a respiratory infection. The test was carried out via multiplex PCR using an in-house method, as described by Bonzel et al (PMID: 18520973). |
| Respiratory viruses | KI | All units at the hospital | Influenza, RSV, and SARS-CoV-2 are included in a multiplex-PCR. Multiplex PCR testing from GeneXpert Dx and Qiastat-Dx Analyzer was mainly used. A more comprehensive viral respiratory panel is also used when infections with other respiratory viruses is suspected. |
| Respiratory viruses | PUM | All units at the hospital | Included in a comprehensive multiplex respiratory PCR panel from Qiastat Dx Analyzer (Qiagen, Germany) according to the manufacturer protocol. These assays are only routinely performed in patients with COVID-19 if a superinfection with another bacterial or viral agent is suspected. They are conducted sporadically in patients with confirmed COVID-19. |
| Respiratory viruses | VULSK | All units at the hospital | Very low testing during the first waves of the COVID-19 patients. Performed only in severely ill patients. When suspected a co-infection Influenza, RSV, and SARS-CoV-2 multiplex PCR from GeneXpert Dx was mostly used. |

**Abbreviations:** Ag=Antigen, BAL=Bronchoalveolar lavage, BAS=Bronchial aspirate, COVID-19=Coronavirus disease 2019, ICU=Intensive care unit, LRT=Lower respiratory tract , LRTI=Lower respiratory tract infection, PCR=Polymerase chain reactions

**Table S2. Classification of contaminants and pathogens**

| **Test modality** | **Organism** | **Classification** | **Number of individuals** |
| --- | --- | --- | --- |
| Blood culture | Achromobacter xylosoxidans | Contaminant | 2 |
| Blood culture | Acinetobacter baumanii | Pathogen | 1 |
| Blood culture | Acinetobacter species | Contaminant | 7 |
| Blood culture | Actinomyces neuii | Contaminant | 5 |
| Blood culture | Actinomyces odontolyticus | Contaminant | 3 |
| Blood culture | Actinomyces species | Contaminant | 2 |
| Blood culture | Actinotignum schaalii | Pathogen | 7 |
| Blood culture | Actinotignum species | Pathogen | 4 |
| Blood culture | Aerobic spores | Contaminant | 1 |
| Blood culture | Aerococcus sanguinicola | Pathogen | 3 |
| Blood culture | Aerococcus species | Pathogen | 1 |
| Blood culture | Aerococcus urinae | Pathogen | 11 |
| Blood culture | Aeromonas species | Contaminant | 2 |
| Blood culture | Aggregatibacter aphrophilus | Contaminant | 4 |
| Blood culture | Anaerobic mixed flora | Contaminant | 1 |
| Blood culture | Bacillus cereus | Contaminant | 3 |
| Blood culture | Bacillus clausii | Contaminant | 1 |
| Blood culture | Bacillus licheniformis | Contaminant | 1 |
| Blood culture | Bacillus species | Contaminant | 5 |
| Blood culture | Bacillus thuringiensis | Contaminant | 1 |
| Blood culture | Bacteroides fragilis | Pathogen | 18 |
| Blood culture | Bacteroides species | Pathogen | 1 |
| Blood culture | Bifidobacterium species | Contaminant | 1 |
| Blood culture | Brachybacterium species | Contaminant | 1 |
| Blood culture | Brevibacillus species | Contaminant | 1 |
| Blood culture | Brevibacterium casei | Contaminant | 1 |
| Blood culture | Brevibacterium species | Contaminant | 3 |
| Blood culture | Campylobacter coli | Pathogen | 1 |
| Blood culture | Campylobacter species | Contaminant | 1 |
| Blood culture | Campylobacter ureolyticus | Contaminant | 1 |
| Blood culture | Candida albicans | Pathogen | 4 |
| Blood culture | Candida dubliniensis | Pathogen | 2 |
| Blood culture | Candida glabrata | Pathogen | 3 |
| Blood culture | Candida parapsilosis | Pathogen | 1 |
| Blood culture | Candida species | Pathogen | 1 |
| Blood culture | Cardiobacterium species | Contaminant | 2 |
| Blood culture | Cellulomonas species | Contaminant | 1 |
| Blood culture | Citrobacter freundii | Pathogen | 6 |
| Blood culture | Citrobacter koseri | Pathogen | 7 |
| Blood culture | Citrobacter species | Pathogen | 2 |
| Blood culture | Clostridium perfringens | Pathogen | 8 |
| Blood culture | Clostridium ramosum | Pathogen | 4 |
| Blood culture | Clostridium species | Contaminant | 2 |
| Blood culture | Coagulase negative Staphylococci | Contaminant | 906 |
| Blood culture | Contamination | Contaminant | 29 |
| Blood culture | Corynebacterium amycolatum | Contaminant | 2 |
| Blood culture | Corynebacterium minutissimum | Contaminant | 1 |
| Blood culture | Corynebacterium species | Contaminant | 41 |
| Blood culture | Corynebacterium urealyticum | Contaminant | 1 |
| Blood culture | Cutibacterium acnes | Contaminant | 72 |
| Blood culture | Cutibacterium species | Contaminant | 2 |
| Blood culture | Dermabacter hominis | Contaminant | 5 |
| Blood culture | Dermabacter species | Contaminant | 4 |
| Blood culture | Dialister species | Contaminant | 2 |
| Blood culture | Dietzia species | Contaminant | 1 |
| Blood culture | Eggerthella lenta | Contaminant | 3 |
| Blood culture | Eikenella corrodens | Pathogen | 2 |
| Blood culture | Eikenella species | Contaminant | 1 |
| Blood culture | Enterobacter cloacae | Pathogen | 23 |
| Blood culture | Enterobacter species | Pathogen | 2 |
| Blood culture | Enterococcus avium | Pathogen | 1 |
| Blood culture | Enterococcus casseliflavus | Pathogen | 3 |
| Blood culture | Enterococcus faecalis | Pathogen | 55 |
| Blood culture | Enterococcus faecium | Pathogen | 38 |
| Blood culture | Enterococcus hirae | Pathogen | 1 |
| Blood culture | Escherichia coli | Pathogen | 349 |
| Blood culture | Eubacterium species | Contaminant | 1 |
| Blood culture | Exiguobacterium species | Contaminant | 1 |
| Blood culture | Filifactor alocis | Contaminant | 1 |
| Blood culture | Fusobacterium nucleatum | Contaminant | 3 |
| Blood culture | Fusobacterium species | Contaminant | 2 |
| Blood culture | Gemella species | Contaminant | 2 |
| Blood culture | Globicatella species | Pathogen | 3 |
| Blood culture | Gordonia species | Contaminant | 1 |
| Blood culture | Gram negative coccus | Contaminant | 1 |
| Blood culture | Gram negative rod | Contaminant | 5 |
| Blood culture | Gram positive rod | Contaminant | 13 |
| Blood culture | Gram positive rods | Contaminant | 1 |
| Blood culture | Granulicatella adiacens | Contaminant | 5 |
| Blood culture | Growth of organism | Contaminant | 13 |
| Blood culture | Haemophilus influenzae | Pathogen | 5 |
| Blood culture | Haemophilus paraphrohaemolyticus | Pathogen | 2 |
| Blood culture | Hafnia alvei | Contaminant | 1 |
| Blood culture | Hungatella hathewayi | Contaminant | 1 |
| Blood culture | Hungatella species | Contaminant | 4 |
| Blood culture | Klebsiella aerogenes | Pathogen | 4 |
| Blood culture | Klebsiella oxytoca | Pathogen | 28 |
| Blood culture | Klebsiella pneumoniae | Pathogen | 82 |
| Blood culture | Klebsiella variicola | Pathogen | 9 |
| Blood culture | Kocuria species | Contaminant | 3 |
| Blood culture | Lactobacillus casei | Contaminant | 5 |
| Blood culture | Lactobacillus species | Contaminant | 3 |
| Blood culture | Lancefieldella species | Contaminant | 2 |
| Blood culture | Leuconostoc species | Contaminant | 3 |
| Blood culture | Listeria monocytogenes | Pathogen | 6 |
| Blood culture | Lysinibacillus sphaericus | Contaminant | 2 |
| Blood culture | Micrococcus luteus | Contaminant | 10 |
| Blood culture | Micrococcus species | Contaminant | 19 |
| Blood culture | Mixed bacterial flora | Contaminant | 9 |
| Blood culture | Moraxella species | Contaminant | 5 |
| Blood culture | Morganella morganii | Pathogen | 3 |
| Blood culture | Mycobacterium avium | Pathogen | 2 |
| Blood culture | Mycobacterium species | Contaminant | 1 |
| Blood culture | Neisseria species | Contaminant | 2 |
| Blood culture | Paenibacillus species | Contaminant | 2 |
| Blood culture | Pantoea species | Contaminant | 5 |
| Blood culture | Parvimonas micra | Contaminant | 4 |
| Blood culture | Peptoniphilus species | Contaminant | 2 |
| Blood culture | Prevotella species | Contaminant | 4 |
| Blood culture | Proteus mirabilis | Pathogen | 27 |
| Blood culture | Proteus vulgaris | Pathogen | 4 |
| Blood culture | Providencia rettgeri | Contaminant | 3 |
| Blood culture | Pseudoglutamicibacter cumminsii | Contaminant | 4 |
| Blood culture | Pseudoglutamicibacter species | Contaminant | 1 |
| Blood culture | Pseudomonas aeruginosa | Pathogen | 33 |
| Blood culture | Pseudomonas mendocina | Contaminant | 1 |
| Blood culture | Pseudomonas putida | Contaminant | 1 |
| Blood culture | Psychrobacter species | Contaminant | 1 |
| Blood culture | Raoultella ornithinolytica | Contaminant | 1 |
| Blood culture | Roseomonas mucosa | Contaminant | 1 |
| Blood culture | Rothia mucilaginosa | Contaminant | 2 |
| Blood culture | Rothia species | Contaminant | 2 |
| Blood culture | Ruminococcus species | Contaminant | 1 |
| Blood culture | Salmonella Stanley | Pathogen | 1 |
| Blood culture | Salmonella Typhi | Pathogen | 6 |
| Blood culture | Salmonella braenderup | Pathogen | 1 |
| Blood culture | Salmonella dublin | Pathogen | 1 |
| Blood culture | Serratia marcescens | Pathogen | 8 |
| Blood culture | Slackia exigua | Contaminant | 1 |
| Blood culture | Sphingomonas paucimobilis | Contaminant | 1 |
| Blood culture | Sporolactobacillus species | Contaminant | 1 |
| Blood culture | Staphylococcus argenteus | Contaminant | 1 |
| Blood culture | Staphylococcus aureus | Pathogen | 204 |
| Blood culture | Staphylococcus auricularis | Contaminant | 1 |
| Blood culture | Staphylococcus capitis | Contaminant | 14 |
| Blood culture | Staphylococcus caprae | Contaminant | 1 |
| Blood culture | Staphylococcus epidermidis | Contaminant | 546 |
| Blood culture | Staphylococcus haemoliticus | Contaminant | 6 |
| Blood culture | Staphylococcus haemolyticus | Contaminant | 2 |
| Blood culture | Staphylococcus hominis | Contaminant | 61 |
| Blood culture | Staphylococcus lugdunensis | Contaminant | 12 |
| Blood culture | Staphylococcus pettenkoferi | Contaminant | 2 |
| Blood culture | Staphylococcus saccharolyticus | Contaminant | 2 |
| Blood culture | Staphylococcus species | Contaminant | 1 |
| Blood culture | Staphylococcus warneri | Contaminant | 2 |
| Blood culture | Stenotrophomonas maltophilia | Pathogen | 2 |
| Blood culture | Streptococci, Alpha | Pathogen | 1 |
| Blood culture | Streptococci, beta-hemolytic group C | Pathogen | 9 |
| Blood culture | Streptococci, beta-hemolytic group G | Pathogen | 16 |
| Blood culture | Streptococcus agalactiae | Pathogen | 21 |
| Blood culture | Streptococcus anginosus | Pathogen | 40 |
| Blood culture | Streptococcus bovis | Pathogen | 9 |
| Blood culture | Streptococcus dysgalactiae | Pathogen | 1 |
| Blood culture | Streptococcus mitis | Pathogen | 2 |
| Blood culture | Streptococcus mitis/oralis | Pathogen | 2 |
| Blood culture | Streptococcus mitis/sanguinis | Pathogen | 41 |
| Blood culture | Streptococcus mutans | Pathogen | 3 |
| Blood culture | Streptococcus parasanguinis | Pathogen | 1 |
| Blood culture | Streptococcus pneumoniae | Pathogen | 72 |
| Blood culture | Streptococcus pyogenes | Pathogen | 8 |
| Blood culture | Streptococcus salivarius | Pathogen | 21 |
| Blood culture | Streptococcus sanguinis | Pathogen | 3 |
| Blood culture | Streptococcus species | Pathogen | 1 |
| Blood culture | Turicella otitidis | Contaminant | 1 |
| Blood culture | Veillonella species | Contaminant | 8 |
| LRT culture | Achromobacter xylosoxidans | Contaminant | 3 |
| LRT culture | Acinetobacter pittii | Contaminant | 1 |
| LRT culture | Aeromonas caviae | Contaminant | 1 |
| LRT culture | Airway flora | Contaminant | 1219 |
| LRT culture | Aspergillus flavus | Contaminant | 1 |
| LRT culture | Candida albicans | Contaminant | 15 |
| LRT culture | Candida glabrata | Contaminant | 2 |
| LRT culture | Candida species | Contaminant | 13 |
| LRT culture | Candida tropicalis | Contaminant | 1 |
| LRT culture | Chryseobacterium indologenes | Contaminant | 1 |
| LRT culture | Citrobacter koseri | Pathogen | 3 |
| LRT culture | Clostridium perfringens | Contaminant | 2 |
| LRT culture | Clostridium species | Contaminant | 1 |
| LRT culture | Contamination | Contaminant | 1 |
| LRT culture | Corynebacterium pseudodiphtheriticum | Contaminant | 1 |
| LRT culture | Dermatophyte | Contaminant | 1 |
| LRT culture | Enterobacter cloacae | Pathogen | 8 |
| LRT culture | Enterococcus faecalis | Contaminant | 1 |
| LRT culture | Escherichia coli | Pathogen | 15 |
| LRT culture | Gram negative mixed flora | Contaminant | 4 |
| LRT culture | Haemophilus influenzae | Pathogen | 116 |
| LRT culture | Hafnia alvei | Pathogen | 1 |
| LRT culture | Klebsiella aerogenes | Pathogen | 4 |
| LRT culture | Klebsiella oxytoca | Pathogen | 13 |
| LRT culture | Klebsiella pneumoniae | Pathogen | 19 |
| LRT culture | Legionella bozemanae | Pathogen | 1 |
| LRT culture | Moraxella catarrhalis | Pathogen | 43 |
| LRT culture | Mycobacterium avium | Pathogen | 2 |
| LRT culture | Mycobacterium chimaera | Pathogen | 3 |
| LRT culture | Mycobacterium gordonae | Pathogen | 1 |
| LRT culture | Mycobacterium intracellulare | Pathogen | 3 |
| LRT culture | Mycobacterium species | Pathogen | 5 |
| LRT culture | Mycobacterium tuberculosis | Pathogen | 4 |
| LRT culture | Neisseria meningitidis | Pathogen | 4 |
| LRT culture | Normal bacterial flora | Contaminant | 2 |
| LRT culture | Pasteurella canis | Contaminant | 1 |
| LRT culture | Proteus mirabilis | Pathogen | 8 |
| LRT culture | Pseudomonas aeruginosa | Pathogen | 52 |
| LRT culture | Robinsoniella peoriensis | Pathogen | 1 |
| LRT culture | Serratia marcescens | Pathogen | 6 |
| LRT culture | Staphylococcus aureus | Pathogen | 114 |
| LRT culture | Stenotrophomonas maltophilia | Pathogen | 7 |
| LRT culture | Streptococci, beta-hemolytic group C or G | Pathogen | 1 |
| LRT culture | Streptococcus agalactiae | Pathogen | 2 |
| LRT culture | Streptococcus pneumoniae | Pathogen | 75 |
| LRT culture | Streptococcus pyogenes | Pathogen | 2 |
| LRT culture | Yeast | Contaminant | 44 |
| Respiratory bacterial DNA assay | Chlamydophila species | Pathogen | 3 |
| Respiratory bacterial DNA assay | Legionella pneumophila | Pathogen | 1 |
| Respiratory bacterial DNA assay | Legionella species | Pathogen | 1 |
| Respiratory bacterial DNA assay | Mycoplasma pneumoniae | Pathogen | 12 |
| Respiratory virus | Adenovirus | Pathogen | 3 |
| Respiratory virus | Coronavirus 229E | Pathogen | 1 |
| Respiratory virus | Coronavirus OC43 | Pathogen | 1 |
| Respiratory virus | Enterovirus | Pathogen | 1 |
| Respiratory virus | Enterovirus D68 | Pathogen | 1 |
| Respiratory virus | Influenza A | Pathogen | 60 |
| Respiratory virus | Influenza B | Pathogen | 7 |
| Respiratory virus | Metapneumovirus | Pathogen | 2 |
| Respiratory virus | Parainfluenzavirus 2 | Pathogen | 1 |
| Respiratory virus | Parainfluenzavirus 3 | Pathogen | 2 |
| Respiratory virus | RSV | Pathogen | 46 |
| Respiratory virus | Respiratory virus | Pathogen | 2 |
| Respiratory virus | Rhinovirus | Pathogen | 10 |
| Urinary antigen | Legionella pneumophila | Pathogen | 10 |
| Urinary antigen | Legionella species | Pathogen | 6 |
| Urinary antigen | Streptococcus pneumoniae | Pathogen | 102 |

**Abbreviations:** LRT=Lower respiratory tract, RSV=Respiratory syncytial virus

**Figure S2. Study flow chart**

**
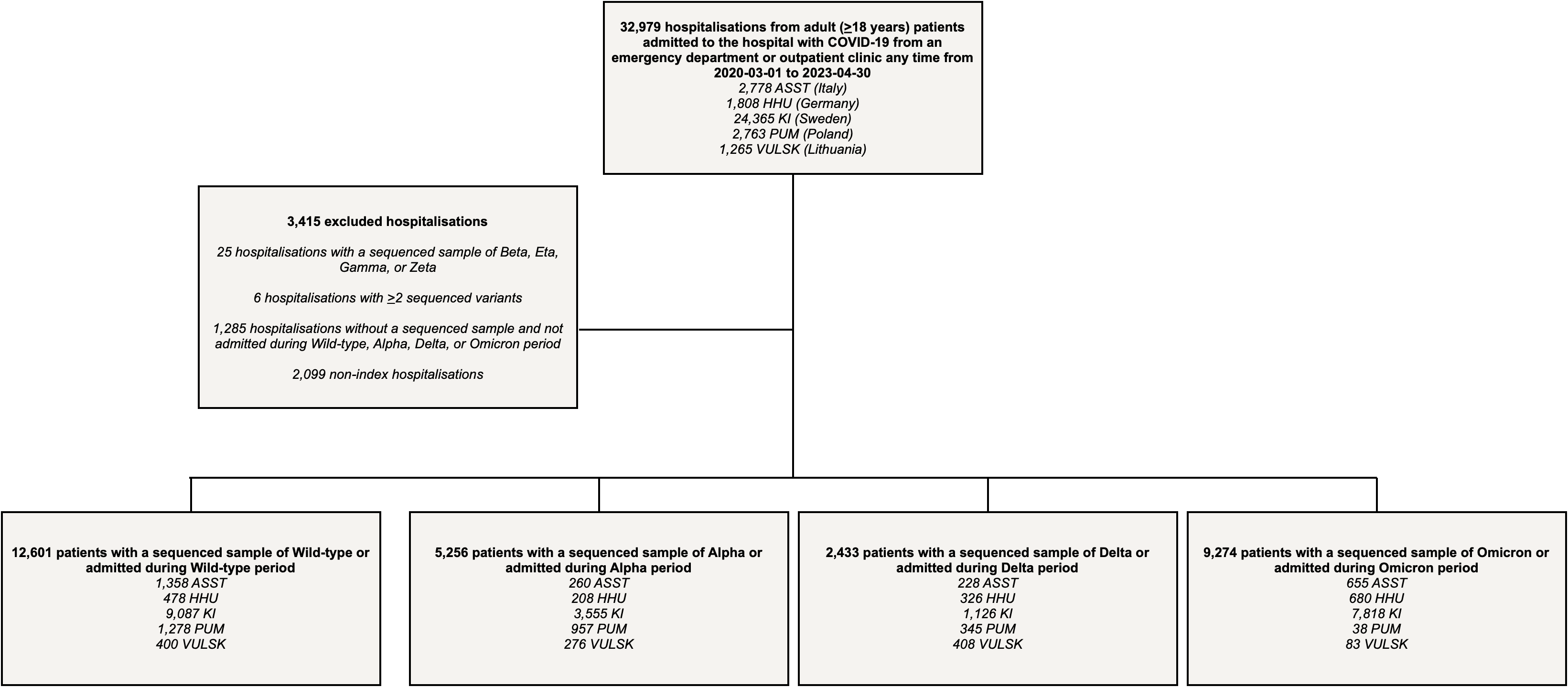
**

**Abbreviations:** COVID-19=Coronavirus disease 2019

**Table S3. Characteristics of the study population by centre**

| **Variable** | **ASST (n=2,501)** | **HHU (n=1,692)** | **KI (n=21,586)** | **PUM (n=2,618)** | **VULSK (n=1,167)** | ***P* value** |
| --- | --- | --- | --- | --- | --- | --- |
| Age, years, median [IQR] | 72.0 [56.0, 83.0] | 60.0 [44.0, 74.0] | 68.0 [54.0, 80.0] | 66.0 [55.0, 74.0] | 55.0 [44.0, 65.0] | <0.001 |
| Age category, years, n (%) |  |  |  |  |  | <0.001 |
| 18-39 | 206 (8.2) | 329 (19.4) | 1,979 (9.2) | 175 (6.7) | 190 (16.3) |  |
| 40-49 | 217 (8.7) | 206 (12.2) | 2,104 (9.7) | 288 (11.0) | 254 (21.8) |  |
| 50-59 | 350 (14.0) | 281 (16.6) | 3,307 (15.3) | 394 (15.0) | 273 (23.4) |  |
| 60-69 | 364 (14.6) | 331 (19.6) | 3,795 (17.6) | 747 (28.5) | 288 (24.7) |  |
| 70-79 | 532 (21.3) | 261 (15.4) | 4,704 (21.8) | 597 (22.8) | 116 (9.9) |  |
| 80 years or older | 832 (33.3) | 284 (16.8) | 5,697 (26.4) | 417 (15.9) | 46 (3.9) |  |
| Male sex, n (%) | 1,440 (57.6) | 940 (55.6) | 12,467 (57.8) | 1,489 (56.9) | 659 (56.5) | 0.39 |
| Comorbidities, n (%) |  |  |  |  |  |  |
| Cancer | 203 (8.1) | 138 (8.2) | 1,987 (9.2) | 118 (4.5) | 32 (2.7) | <0.001 |
| Cardiac or cerebrovascular disease | 711 (28.4) | 569 (33.6) | 7,050 (32.7) | 217 (8.3) | 159 (13.6) | <0.001 |
| Chronic kidney disease | 214 (8.6) | 608 (35.9) | 2,608 (12.1) | 48 (1.8) | 188 (16.1) | <0.001 |
| Chronic liver disease | 86 (3.4) | 75 (4.4) | 588 (2.7) | 27 (1.0) | 14 (1.2) | <0.001 |
| Chronig lung disease | 334 (13.4) | 138 (8.2) | 4,373 (20.3) | 7 (0.3) | 47 (4.0) | <0.001 |
| Diabetes | 480 (19.2) | 337 (19.9) | 5,069 (23.5) | 269 (10.3) | 173 (14.8) | <0.001 |
| Hypertension | 1,226 (49.0) | 739 (43.7) | 10,620 (49.2) | 588 (22.5) | 334 (28.6) | <0.001 |
| Immunocompromised | 56 (2.2) | 375 (22.2) | 2,901 (13.4) | 18 (0.7) | 8 (0.7) | <0.001 |
| Neurologic conditions | 252 (10.1) | 343 (20.3) | 1,970 (9.1) | 10 (0.4) | 28 (2.4) | <0.001 |
| Obesity | 305 (12.2) | 55 (3.3) | 5,064 (23.5) | 42 (1.6) | 64 (5.5) | <0.001 |
| Number of comorbidities, n (%) |  |  |  |  |  | <0.001 |
| 0 | 668 (26.7) | 359 (21.2) | 4,739 (22.0) | 1,660 (63.4) | 586 (50.2) |  |
| 1 | 678 (27.1) | 342 (20.2) | 4,839 (22.4) | 629 (24.0) | 300 (25.7) |  |
| 2 | 590 (23.6) | 375 (22.2) | 4,449 (20.6) | 279 (10.7) | 144 (12.3) |  |
| 3 | 338 (13.5) | 319 (18.9) | 3,755 (17.4) | 43 (1.6) | 95 (8.1) |  |
| 4 or more | 227 (9.1) | 297 (17.6) | 3,804 (17.6) | 7 (0.3) | 42 (3.6) |  |
| COVID-19 vaccination doses, n (%)^a^ |  |  |  |  |  | <0.001 |
| Unvaccinated | 2,090 (83.7) | 599 (49.0) | 14,120 (65.4) | 2,481 (94.8) | 1,138 (97.5) |  |
| 1 dose | 36 (1.4) | 20 (1.6) | 453 (2.1) | 122 (4.7) | 2 (0.2) |  |
| 2 doses | 231 (9.3) | 176 (14.4) | 1,776 (8.2) | 14 (0.5) | 16 (1.4) |  |
| 3 doses or more | 140 (5.6) | 428 (35.0) | 5,237 (24.3) | 1 (0.0) | 11 (0.9) |  |
| SARS-CoV-2 variant period |  |  |  |  |  | <0.001 |
| Wild-type | 1,358 (54.3) | 478 (28.3) | 9,087 (42.1) | 1,278 (48.8) | 400 (34.3) |  |
| Alpha | 260 (10.4) | 208 (12.3) | 3,555 (16.5) | 957 (36.6) | 276 (23.7) |  |
| Delta | 228 (9.1) | 326 (19.3) | 1,126 (5.2) | 345 (13.2) | 408 (35.0) |  |
| Omicron | 655 (26.2) | 680 (40.2) | 7,818 (36.2) | 38 (1.5) | 83 (7.1) |  |
| Hospital length of stay, days, median [IQR] | 10.0 [6.0, 18.0] | 6.0 [3.0, 13.0] | 5.0 [3.0, 9.0] | 12.0 [8.0, 17.0] | 10.0 [8.0, 15.0] | <0.001 |
| ICU admission, n (%)^b^ | 69 (3.1) | 409 (24.2) | 2,123 (9.8) | 162 (6.2) | 57 (4.9) | <0.001 |
| ICU length of stay, days, median [IQR]^b^ | 9.0 [5.0, 18.0] | 1.0 [0.0, 5.0] | 5.0 [2.0, 11.0] | 12.0 [6.0, 22.8] | 6.0 [3.0, 9.0] | <0.001 |
| In-hospital mortality, n (%) | 531 (21.2) | 198 (11.7) | 2,063 (9.6) | 423 (16.2) | 59 (5.1) | <0.001 |

a. 473 patients were excluded due to missing data

b. 294 patients were excluded due to missing data

Note: Kruskal-Wallis tests were used for continuous variables and Chi squared tests were used for categorical variables.

**Abbreviations:** COVID-19=Coronavirus disease 2019, ICU=Intensive care unit, IQR=Interquartile range, SARS-CoV-2=Severe acute respiratory syndrome coronavirus 2

**Figure S3. Testing frequencies and positivity rates per centre**

**
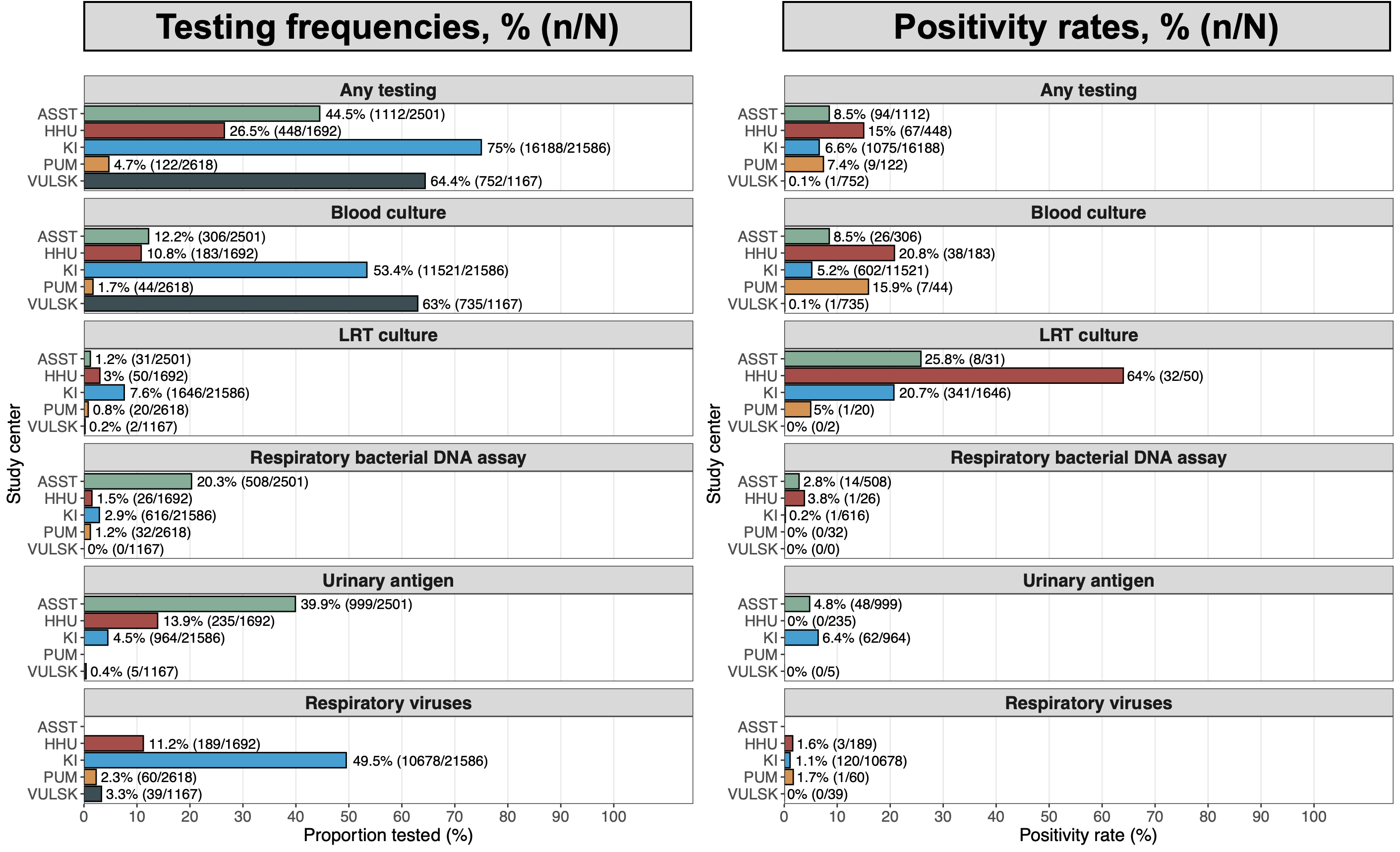
**

**Notes:** PUM were excluded from the analyses of urinary antigen testing due to lack of coverage in the data sources. ASST were excluded from the analyses of respiratory viruses due to lack of coverage in the data sources.

**Abbreviations:** LRT=Lower respiratory tract

**Table S4. The most commonly identified organisms per SARS-CoV-2 variant**

| **SARS-CoV-2 variant** | **Identified organism** | **Number of individuals** |
| --- | --- | --- |
| Wild type | Staphylococcus aureus | 87 |
| Wild type | Escherichia coli | 48 |
| Wild type | Streptococcus pneumoniae | 45 |
| Wild type | Haemophilus influenzae | 23 |
| Wild type | Klebsiella pneumoniae | 23 |
| Wild type | Pseudomonas aeruginosa | 18 |
| Wild type | Moraxella catarrhalis | 12 |
| Wild type | Mycoplasma pneumoniae | 11 |
| Wild type | Klebsiella oxytoca | 8 |
| Wild type | Enterococcus faecium | 7 |
| Wild type | Proteus mirabilis | 7 |
| Alpha | Staphylococcus aureus | 31 |
| Alpha | Streptococcus pneumoniae | 18 |
| Alpha | Haemophilus influenzae | 12 |
| Alpha | Pseudomonas aeruginosa | 10 |
| Alpha | Escherichia coli | 9 |
| Alpha | Moraxella catarrhalis | 7 |
| Alpha | Streptococcus salivarius | 6 |
| Alpha | Streptococcus mitis/sanguinis | 4 |
| Alpha | Klebsiella pneumoniae | 3 |
| Alpha | Enterobacter cloacae | 2 |
| Alpha | Enterococcus faecalis | 2 |
| Alpha | Enterococcus faecium | 2 |
| Alpha | Klebsiella oxytoca | 2 |
| Alpha | Stenotrophomonas maltophilia | 2 |
| Delta | Streptococcus pneumoniae | 16 |
| Delta | Escherichia coli | 11 |
| Delta | Staphylococcus aureus | 10 |
| Delta | Haemophilus influenzae | 8 |
| Delta | Moraxella catarrhalis | 6 |
| Delta | Enterobacter cloacae | 4 |
| Delta | Klebsiella oxytoca | 3 |
| Delta | Pseudomonas aeruginosa | 3 |
| Delta | Streptococcus anginosus | 3 |
| Delta | Streptococcus mitis/sanguinis | 3 |
| Omicron | Escherichia coli | 141 |
| Omicron | Streptococcus pneumoniae | 114 |
| Omicron | Staphylococcus aureus | 93 |
| Omicron | Haemophilus influenzae | 63 |
| Omicron | Influenza A | 51 |
| Omicron | Klebsiella pneumoniae | 40 |
| Omicron | RSV | 38 |
| Omicron | Pseudomonas aeruginosa | 35 |
| Omicron | Enterococcus faecalis | 25 |
| Omicron | Streptococcus mitis/sanguinis | 23 |

**Abbreviations:** RSV=Respiratory syncytial virus, SARS-CoV-2=Severe acute respiratory syndrome coronavirus 2

**Table S5. The most commonly identified organisms per SARS-CoV-2 variant among patients from KI with a main diagnosis of COVID-19**

| **SARS-CoV-2 variant** | **Identified organism** | **Number of individuals** |
| --- | --- | --- |
| Wild type | Staphylococcus aureus | 57 |
| Wild type | Escherichia coli | 26 |
| Wild type | Streptococcus pneumoniae | 21 |
| Wild type | Haemophilus influenzae | 18 |
| Wild type | Moraxella catarrhalis | 12 |
| Wild type | Pseudomonas aeruginosa | 12 |
| Wild type | Klebsiella pneumoniae | 8 |
| Wild type | Legionella pneumophila | 4 |
| Wild type | Streptococcus salivarius | 4 |
| Wild type | RSV | 3 |
| Wild type | Streptococcus anginosus | 3 |
| Wild type | Streptococcus bovis | 3 |
| Wild type | Streptococcus mitis/sanguinis | 3 |
| Wild type | Streptococcus sanguinis | 3 |
| Alpha | Staphylococcus aureus | 21 |
| Alpha | Haemophilus influenzae | 12 |
| Alpha | Streptococcus pneumoniae | 11 |
| Alpha | Moraxella catarrhalis | 6 |
| Alpha | Streptococcus salivarius | 5 |
| Alpha | Pseudomonas aeruginosa | 3 |
| Alpha | Streptococcus mitis/sanguinis | 3 |
| Alpha | Aerococcus urinae | 1 |
| Alpha | Bacteroides fragilis | 1 |
| Alpha | Escherichia coli | 1 |
| Alpha | Klebsiella pneumoniae | 1 |
| Alpha | Neisseria meningitidis | 1 |
| Delta | Haemophilus influenzae | 7 |
| Delta | Staphylococcus aureus | 7 |
| Delta | Streptococcus pneumoniae | 7 |
| Delta | Moraxella catarrhalis | 5 |
| Delta | Escherichia coli | 2 |
| Delta | Klebsiella pneumoniae | 2 |
| Delta | Bacteroides fragilis | 1 |
| Delta | Citrobacter koseri | 1 |
| Delta | Enterobacter cloacae | 1 |
| Delta | Klebsiella aerogenes | 1 |
| Delta | Klebsiella oxytoca | 1 |
| Delta | Listeria monocytogenes | 1 |
| Delta | Pseudomonas aeruginosa | 1 |
| Delta | RSV | 1 |
| Delta | Salmonella dublin | 1 |
| Delta | Streptococcus anginosus | 1 |
| Delta | Streptococcus mitis/sanguinis | 1 |
| Delta | Streptococcus salivarius | 1 |
| Omicron | Streptococcus pneumoniae | 42 |
| Omicron | Haemophilus influenzae | 33 |
| Omicron | Staphylococcus aureus | 28 |
| Omicron | RSV | 14 |
| Omicron | Influenza A | 13 |
| Omicron | Pseudomonas aeruginosa | 11 |
| Omicron | Moraxella catarrhalis | 9 |
| Omicron | Escherichia coli | 7 |
| Omicron | Streptococcus mitis/sanguinis | 7 |
| Omicron | Klebsiella pneumoniae | 5 |

**Abbreviations:** RSV=Respiratory syncytial virus, SARS-CoV-2=Severe acute respiratory syndrome coronavirus 2

**Table S6. Subdistribution hazard ratios for 28-day in-hospital mortality and risk ratios for in-hospital mortality by co-infection status for the overall study population as well as pre-defined subgroups**

| **Group** | **Unadjusted risk ratio**  **(95% CI)** | **Unadjusted subdistribution hazard ratio**  **(95% CI)** | **Adjusted risk ratio**  **(95% CI)^a^** | **Adjusted subdistribution hazard ratio**  **(95% CI)** |
| --- | --- | --- | --- | --- |
| Overall | *Reference* | *Reference* | *Reference^b^* | *Reference^b^* |
| Overall | 1.73 (1.54-1.96) | 1.86 (1.62-2.14) | 1.69 (1.49-1.91) | 1.82 (1.56-2.11) |
| Pre-Omicron | *Reference* | *Reference* | *Reference^c^* | *Reference^c^* |
| Pre-Omicron | 2.16 (1.86-2.51) | 2.40 (2.01-2.88) | 1.58 (1.36-1.84) | 1.71 (1.40-2.09) |
| Omicron | *Reference* | *Reference* | *Reference^d^* | *Reference^d^* |
| Omicron | 1.90 (1.55-2.33) | 1.98 (1.58-2.47) | 1.87 (1.52-2.30) | 1.94 (1.54-2.45) |
| Tested patients | *Reference* | *Reference* | *Reference^b^* | *Reference^b^* |
| Tested patients | 1.63 (1.44-1.85) | 1.73 (1.50-2.00) | 1.53 (1.35-1.73) | 1.60 (1.38-1.87) |
| KI only | *Reference* | *Reference* | *Reference^e^* | *Reference^e^* |
| KI only | 1.79 (1.55-2.06) | 1.89 (1.61-2.22) | 1.71 (1.49-1.97) | 1.84 (1.55-2.19) |
| KI only, COVID-19 main diagnosis | *Reference* | *Reference* | *Reference^e^* | *Reference^e^* |
| KI only, COVID-19 main diagnosis | 1.83 (1.51-2.21) | 1.92 (1.53-2.40) | 1.53 (1.26-1.85) | 1.60 (1.25-2.04) |
| All centres except KI | *Reference* | *Reference* | *Reference^f^* | *Reference^f^* |
| All centres except KI | 2.17 (1.74-2.72) | 2.47 (1.86-3.27) | 1.51 (1.18-1.95) | 1.61 (1.14-2.26) |
| <70 years | *Reference* | *Reference* | *Reference^g^* | *Reference^g^* |
| <70 years | 2.63 (2.05-3.37) | 3.04 (2.29-4.03) | 2.52 (1.94-3.27) | 3.08 (2.23-4.25) |
| >70 years | *Reference* | *Reference* | *Reference^h^* | *Reference^h^* |
| >70 years | 1.26 (1.10-1.45) | 1.31 (1.12-1.54) | 1.52 (1.33-1.75) | 1.61 (1.36-1.91) |
| Immunocompromised | *Reference* | *Reference* | *Reference^i^* | *Reference^i^* |
| Immunocompromised | 1.08 (0.76-1.54) | 1.11 (0.75-1.65) | 1.07 (0.73-1.56) | 1.07 (0.68-1.66) |
| Not immunocompromised | *Reference* | *Reference* | *Reference^i^* | *Reference^i^* |
| Not immunocompromised | 1.88 (1.66-2.14) | 2.04 (1.76-2.37) | 1.83 (1.61-2.08) | 2.00 (1.70-2.35) |
| Unvaccinated | *Reference* | *Reference* | *Reference^j^* | *Reference^j^* |
| Unvaccinated | 2.06 (1.77-2.40) | 2.24 (1.86-2.69) | 1.62 (1.39-1.89) | 1.74 (1.42-2.12) |
| Vaccinated | *Reference* | *Reference* | *Reference^j^* | *Reference^j^* |
| Vaccinated | 1.69 (1.37-2.09) | 1.76 (1.40-2.21) | 1.73 (1.40-2.14) | 1.81 (1.43-2.29) |
| Bacterial co-infections only | *Reference* | *Reference* | *Reference^b^* | *Reference^b^* |
| Bacterial co-infections only | 1.83 (1.62-2.07) | 1.97 (1.71-2.27) | 1.72 (1.52-1.95) | 1.84 (1.58-2.15) |

a. 473 patients were excluded from this model due to missing data on COVID-19 vaccination

b. Adjusted for age category (18-39, 40-49, 50-59, 60-69, 70-79, >80 years), sex (Male, Female), centre (ASST, HHU, KI, PUM, VULSK), all comorbidities considered in the study respectively (see Table 1), number of comorbidities (0, 1, 2, 3, 4 or more), COVID-19 vaccination status (Unvaccinated, 1 dose, 2 doses, 3 doses or more), SARS-CoV-2 variant (Wild type, Alpha Delta, Omicron), and calendar time.

c. Adjusted for age category (18-39, 40-49, 50-59, 60-69, 70-79, >80 years), sex (Male, Female), centre (ASST, HHU, KI, PUM, VULSK), all comorbidities considered in the study respectively (see Table 1), number of comorbidities (0, 1, 2, 3, 4 or more), COVID-19 vaccination status (Unvaccinated, 1 dose, 2 doses, 3 doses or more), SARS-CoV-2 variant (Wild type, Alpha Delta), and calendar time.

d. Adjusted for age category (18-39, 40-49, 50-59, 60-69, 70-79, >80 years), sex (Male, Female), centre (ASST, HHU, KI, PUM, VULSK), all comorbidities considered in the study respectively (see Table 1), number of comorbidities (0, 1, 2, 3, 4 or more), COVID-19 vaccination status (Unvaccinated, 1 dose, 2 doses, 3 doses or more), and calendar time.

e. Adjusted for age category (18-39, 40-49, 50-59, 60-69, 70-79, >80 years), sex (Male, Female), all comorbidities considered in the study respectively (see Table 1), number of comorbidities (0, 1, 2, 3, 4 or more), COVID-19 vaccination status (Unvaccinated, 1 dose, 2 doses, 3 doses or more), SARS-CoV-2 variant (Wild type, Alpha Delta, Omicron), and calendar time.

f. Adjusted for age category (18-39, 40-49, 50-59, 60-69, 70-79, >80 years), sex (Male, Female), centre (ASST, HHU, PUM, VULSK), all comorbidities considered in the study respectively (see Table 1), number of comorbidities (0, 1, 2, 3, 4 or more), COVID-19 vaccination status (Unvaccinated, 1 dose, 2 doses, 3 doses or more), SARS-CoV-2 variant (Wild type, Alpha Delta, Omicron), and calendar time.

g. Adjusted for age category (18-39, 40-49, 50-59, 60-69), sex (Male, Female), centre (ASST, HHU, KI, PUM, VULSK), all comorbidities considered in the study respectively (see Table 1), number of comorbidities (0, 1, 2, 3, 4 or more), COVID-19 vaccination status (Unvaccinated, 1 dose, 2 doses, 3 doses or more), SARS-CoV-2 variant (Wild type, Alpha Delta, Omicron), and calendar time.

h. Adjusted for age category (70-79, >80 years), sex (Male, Female), centre (ASST, HHU, KI, PUM, VULSK), all comorbidities considered in the study respectively (see Table 1), number of comorbidities (0, 1, 2, 3, 4 or more), COVID-19 vaccination status (Unvaccinated, 1 dose, 2 doses, 3 doses or more), SARS-CoV-2 variant (Wild type, Alpha Delta, Omicron), and calendar time.

i. Adjusted for age category (18-39, 40-49, 50-59, 60-69, 70-79, >80 years), sex (Male, Female), centre (ASST, HHU, KI, PUM, VULSK), all comorbidities considered in the study except immunocompromised state respectively (see Table 1), number of comorbidities (0, 1, 2, 3, 4 or more), COVID-19 vaccination status (Unvaccinated, 1 dose, 2 doses, 3 doses or more), SARS-CoV-2 variant (Wild type, Alpha Delta, Omicron), and calendar time.

j. Adjusted for age category (18-39, 40-49, 50-59, 60-69, 70-79, >80 years), sex (Male, Female), centre (ASST, HHU, KI, PUM, VULSK), all comorbidities considered in the study respectively (see Table 1), number of comorbidities (0, 1, 2, 3, 4 or more), SARS-CoV-2 variant (Wild type, Alpha Delta, Omicron), and calendar time.

**Table S7. Identified pathogens among patients dying in-hospital with a verified co-infection overall as well as during the Omicron period only**

| **Pathogen** | **Overall (n=232)** | **Omicron (n=95)** |
| --- | --- | --- |
| Staphylococcus aureus | 67 (24%) | 20 (17.1%) |
| Streptococcus pneumoniae | 30 (10.8%) | 9 (7.7%) |
| Escherichia coli | 29 (10.4%) | 15 (12.8%) |
| Klebsiella pneumoniae | 20 (7.2%) | 9 (7.7%) |
| Pseudomonas aeruginosa | 14 (5%) | 7 (6%) |
| Streptococcus mitis/sanguinis | 12 (4.3%) | 7 (6%) |
| Enterococcus faecalis | 10 (3.6%) | 7 (6%) |
| Haemophilus influenzae | 8 (2.9%) | 3 (2.6%) |
| Enterococcus faecium | 7 (2.5%) | 3 (2.6%) |
| Streptococcus anginosus | 7 (2.5%) | 5 (4.3%) |
| Proteus mirabilis | 6 (2.2%) | 2 (1.7%) |
| RSV | 6 (2.2%) | 5 (4.3%) |
| Streptococcus salivarius | 6 (2.2%) | 2 (1.7%) |
| Bacteroides fragilis | 5 (1.8%) | 4 (3.4%) |
| Enterobacter cloacae | 5 (1.8%) | 1 (0.9%) |
| Klebsiella oxytoca | 3 (1.1%) | 2 (1.7%) |
| Legionella pneumophila | 3 (1.1%) | 1 (0.9%) |
| Moraxella catarrhalis | 3 (1.1%) | 0 |
| Stenotrophomonas maltophilia | 3 (1.1%) | 0 |
| Streptococcus agalactiae | 3 (1.1%) | 1 (0.9%) |
| Citrobacter freundii | 2 (0.7%) | 2 (1.7%) |
| Influenza A | 2 (0.7%) | 2 (1.7%) |
| Klebsiella aerogenes | 2 (0.7%) | 1 (0.9%) |
| Serratia marcescens | 2 (0.7%) | 1 (0.9%) |
| Streptococci, beta-hemolytic group G | 2 (0.7%) | 1 (0.9%) |
| Streptococcus bovis | 2 (0.7%) | 0 |
| Acinetobacter baumanii | 1 (0.4%) | 0 |
| Actinotignum schaalii | 1 (0.4%) | 1 (0.9%) |
| Actinotignum species | 1 (0.4%) | 0 |
| Aerococcus sanguinicola | 1 (0.4%) | 0 |
| Aerococcus urinae | 1 (0.4%) | 0 |
| Bacteroides species | 1 (0.4%) | 0 |
| Candida glabrata | 1 (0.4%) | 1 (0.9%) |
| Citrobacter koseri | 1 (0.4%) | 1 (0.9%) |
| Enterococcus casseliflavus | 1 (0.4%) | 0 |
| Enterococcus hirae | 1 (0.4%) | 0 |
| Globicatella species | 1 (0.4%) | 1 (0.9%) |
| Hafnia alvei | 1 (0.4%) | 0 |
| Klebsiella variicola | 1 (0.4%) | 0 |
| Legionella species | 1 (0.4%) | 1 (0.9%) |
| Listeria monocytogenes | 1 (0.4%) | 0 |
| Morganella morganii | 1 (0.4%) | 1 (0.9%) |
| Neisseria meningitidis | 1 (0.4%) | 0 |
| Streptococcus mitis/oralis | 1 (0.4%) | 0 |
| Streptococcus pyogenes | 1 (0.4%) | 1 (0.9%) |
| Streptococcus species | 1 (0.4%) | 0 |

**Note:** Since several pathogens could be identified in the same patient, the total numbers in each column are greater than the number of patients dying.
